# Monitoring Progress Towards the Elimination of Hepatitis C as a Public Health Threat in Norway: A Modelling Study Among People Who Inject Drugs and Immigrants

**DOI:** 10.1093/infdis/jiae147

**Published:** 2024-03-27

**Authors:** Robert Whittaker, Jørgen E Midtbø, Hilde Kløvstad

**Affiliations:** Department of Infection Control and Vaccines, Norwegian Institute of Public Health, Oslo, Norway; Department of Method Development and Analytics, Norwegian Institute of Public Health, Oslo, Norway; Department of Infection Control and Vaccines, Norwegian Institute of Public Health, Oslo, Norway

**Keywords:** hepatitis C, public health surveillance, disease elimination, mathematical models, Norway

## Abstract

**Background:**

The global incidence target for the elimination of hepatitis C among people who inject drugs (PWID) is <2/100. In Norway, the hepatitis C epidemic is concentrated in PWID. Immigrants are the second most important risk group for chronic infection. We modelled the incidence of hepatitis C among active PWID, and the prevalence of chronic infection among active PWID, ex-PWID, and immigrants in Norway to 2022.

**Methods:**

We built a stochastic compartmental model, which was informed using data from national data sources, literature, and expert opinion. We report median values with 95% credible intervals (CrI).

**Results:**

The model estimated 30 (95% Crl, 13–52) new infections among active PWID in 2022, or 0.37/100 (95% Crl, 0.17–0.65), down from a peak of 726 (95% Crl, 506–1067) in 2000. Across all groups, the model estimated 3202 (95% Crl, 1273–6601) chronically infected persons in 2022. Results were robust in sensitivity analyses.

**Conclusions:**

Norway provides an example of the feasibility of hepatitis C elimination in a setting with a concentrated epidemic, high coverage of harm reduction services, and no treatment restrictions. Continued momentum is needed to further reduce the transmission and burden of hepatitis C in Norway.

## Teaser

We estimate that Norway, a setting with a concentrated epidemic, high coverage of harm reduction services, and no treatment restrictions, has achieved the global incidence target for the elimination of hepatitis C among people who inject drugs.

Chronic infection with hepatitis C virus (HCV) may cause progressive liver fibrosis and is a leading global cause of liver cirrhosis, cancer, and death [1–3]. However, with the development of safe, effective, and short-lasting direct-acting antiviral (DAA) treatment [4], the elimination of hepatitis C as a public health threat has become a feasible global goal. The World Health Organization (WHO) absolute incidence targets for elimination are <5/100 000 in the general population and <2/100 for people who inject drugs (PWID) by 2030 [5, 6]. While there has been some global progress [5], the Polaris Observatory estimates that only 14 countries are on track to achieve the elimination goal [7]. One of those countries is Norway (population 5.4 million).

As in many Western European countries [8], the hepatitis C epidemic in Norway has been driven by the sharing of needles and other paraphernalia for injecting drugs among PWID, who in Norway have historically predominantly injected heroin. Population-based studies have found an HCV RNA prevalence ≤0.5% in the general population, with injecting drugs the predominant route of transmission among participants of Norwegian origin [9–11]. Surveys among PWID attending low-threshold health and social care services across the 4 largest cities (Oslo, Bergen, Stavanger, Trondheim) found a relatively stable trend in HCV RNA prevalence in the period 2002–2017, ranging from 39% to 60% [12, 13]. During this time, treatment for hepatitis C in Norway was dictated by viral genotype, stage of liver disease, and risk of fibrosis progression. Treatment uptake among PWID was low [14].

The Norwegian national strategy against viral hepatitis was launched in 2016 [15] and supplemented in 2018 [16]. Testing for hepatitis C is free, and recommended for defined risk groups [17]. Several low-threshold and outreach testing services for PWID have been implemented, either stand-alone or integrated into services like opioid substitution therapy (OST) [18–22]. Treatment has been scaled-up since the arrival of interferon-free DAA in 2014, and in February 2018 was made freely available for all diagnosed with HCV infection. National treatment guidelines recommend simplified, integrated treatment pathways (for example, in low-threshold and outreach services, like those referenced above) for those deemed to have difficulty following a more traditional treatment course in specialist care. Treatment uptake has steadily increased [13]. A registry-based study among active PWID in Oslo diagnosed with chronic hepatitis C found that the treatment rate increased from <0.5 per 100 in 2010 to >20 per 100 in 2018 [14]. Furthermore, the coverage of harm reduction services that reduce the risk of HCV infection among PWID (needle and syringe programs [NSP] and OST) in Norway is among the world's highest and exceeds elimination targets [6, 13, 23–25]. Municipalities are legally required to freely provide clean needles and syringes to residents in need [13]. Observed HCV RNA prevalence among PWID has subsequently plummeted to <10% in 2022 [13, 26]. In 2022, for the first time most newly diagnosed cases of hepatitis C (RNA or core antigen positive) were immigrants (52%, compared to 21%–30% in 2016–2021).

The surveillance of hepatitis C incidence is notoriously difficult and mathematical modelling has been recommended to monitor progress towards this elimination target [6, 25, 27]. The WHO guidance for validation of elimination states that “robust mathematical modelling can be used … where (i) at least two country-specific prevalence serosurveys are available, and (ii) programmatic data are sufficiently robust as model inputs” [25]. Previous modelling of the hepatitis C epidemic in Norway was restricted to PWID and predates the national strategies and treatment scale-up [15, 28]. Also, the prevalence of hepatitis C among immigrants to Norway is unknown [13]. We generated updated bespoke estimates of the incidence of HCV infection among active PWID, and the prevalence of chronic hepatitis C among active PWID, ex-PWID, and immigrants, to support monitoring progress towards the elimination of hepatitis C in Norway.

## METHODS

### Model Framework

**Figure 1.**
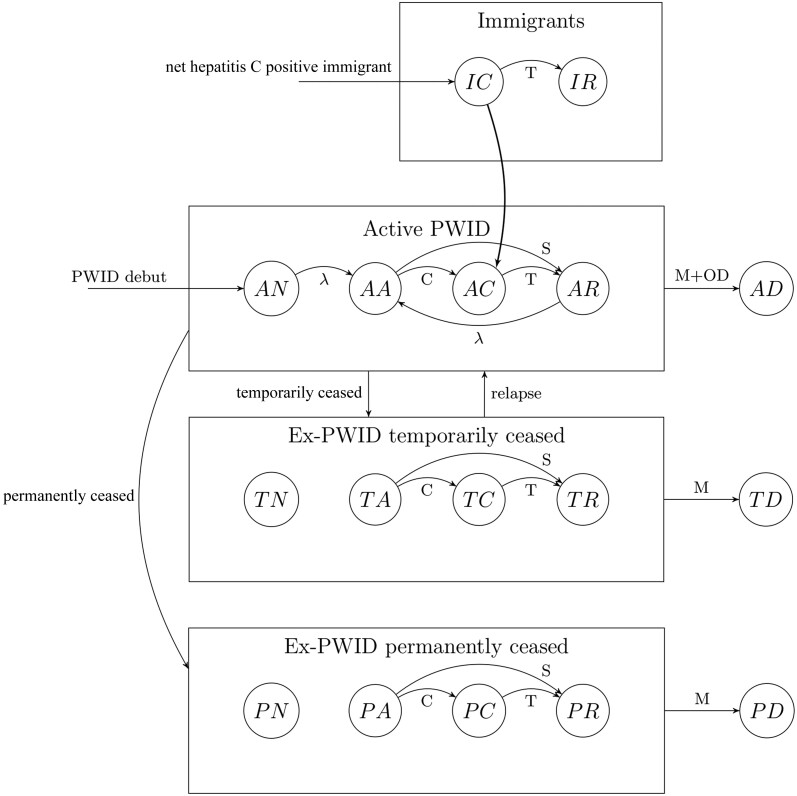
A schematic overview of the stochastic compartmental model to estimate the incidence and prevalence of hepatitis C among people who inject drugs and immigrants in Norway. Abbreviations: AA, active PWID acutely infected with hepatitis C; AC, active PWID chronically infected with hepatitis C; AD, active PWID who has died; AN, active PWID hepatitis C naive; AR, active PWID recovered from hepatitis C infection; C, rate of transition from acutely infected to chronically infected; IC, immigrant chronically infected with hepatitis C; IR, immigrant recovered from hepatitis C infection; M, mortality rate; OD, rate of overdose death; PA, permanently ceased PWID acutely infected with hepatitis C; PC, permanently ceased PWID chronically infected with hepatitis C; PD, permanently ceased PWID who has died; PN, permanently ceased PWID hepatitis C naive; PR, permanently ceased PWID recovered from hepatitis C infection; PWID, people who inject drugs; S, rate of transition from acutely infected to recovered; T, rate of transition from chronically infected to recovered; TA, temporarily ceased PWID acutely infected with hepatitis C; TC, temporarily ceased PWID chronically infected with hepatitis C; TD, temporarily ceased PWID who has died; TN, temporarily ceased PWID hepatitis C naive; TR, temporarily ceased PWID recovered from hepatitis C infection; λ, rate of transition from hepatitis C naive/recovered to acutely infected with hepatitis C.

We built a stochastic compartmental model (Figure 1) to simulate the incidence and prevalence of hepatitis C from 1972 to 2022. For PWID we also made projections until 2030. We modelled from 1972 due to the availability of data on the size of our risk groups, while the late 1960s to early 1970s coincide with the start of the injecting epidemic in Norway and the first of several waves of immigration from the mid-20th century.

We informed the model using data from a range of national data sources, literature, and expert opinion (Table 1). Statistical inference was performed in a Bayesian statistical framework using the particle-Markov chain Monte Carlo (pMCMC) method. Two parameters, namely the rate of debut of new active injectors (excluding the first time step) and rate of hepatitis C treatment, were inferred by allowing them to vary randomly at each time step and particle filtering the resulting trajectories against data to obtain trajectories matching observations. The basic per-person HCV infection rate among active PWID (β) was kept constant through the simulation (albeit modified by geographical dispersion and harm reduction services, see below). Three parameters, namely β, rate of debut of new active injectors at the first time step, and number of active PWID acutely infected at the first time step, were also inferred by the pMCMC. Model outputs came from 2000 simulations, each sampled from the joint posterior distribution on all inferred parameters. We report median values, with credible intervals (CrI) at the 2.5th–97.5th percentile. For derived quantities (eg, comparisons to 2015, the WHO global relative target [25]), the relative difference is calculated for each trajectory before calculating percentiles.

**Table 1.** Summary of Main Data Sources for the Model

Data Element	Data Source to Inform Values For Data Element	Years With Available Data	Range of Values in Model^a^
Size of active PWID, ex-PWID temporarily ceased, and ex-PWID permanently ceased populations	The Norwegian Institute of Public Health, Department of Alcohol, Tobacco, and Drugs [29, 30]	1973–2021^b^	Active: 388 (1973)–11 483 (1999); Ex-PWID temporarily ceased: 10 (1973)–9167 (2003); Ex-PWID permanently ceased: 10 (1973)–7664 (2021)
Active PWID			
Prevalence of hepatitis C to inform the underlying infection rate	Prevalence surveys among PWID attending low-threshold health and social care services in Oslo, Bergen, Stavanger, and Trondheim [12, 13, 22, 26]	2002–2022^c^	6% (Bergen/Stavanger, 2022)–60% (Bergen/Stavanger, 2020)
Rate of spontaneous clearance of acute infections	Systematic review and meta-analysis [31]	—	26%
Coverage of NSP	Centre for Alcohol and Drug Research [24]	2016–2019	77% (2016)–92% (2019)
Effect of NSP on the risk of infection	Systematic review and meta-analysis [32]	—	56%
Number of patients on OST	Norwegian Centre for Addiction Research Centre for Alcohol and Drug Research [21]	1998^d^–2022	204 (1998)–8315 (2022)
Proportion of patients on OST that overlapped with active PWID	Expert opinion, guided by data on self-reported injecting in the last 12 mo and the use of stimulants and illegal opioids in the last 4 wk among OST patients [21]	—	20%
Effect of OST on the risk of infection	Systematic review and meta-analysis [32]	—	50%
County-level data on yearly number of overdose deaths for calculating the Gini coefficient	Norwegian Cause of Death Registry [33]	1996–2022	0 (several counties and years)–113 (Oslo, 1998)
Rate of overdose deaths	Norwegian Cause of Death Registry [33, 34]	1996–2022	0.03 per person per year
Immigrants			
Net annual immigration by country	Statistics Norway [35]	1970–2022	0 (several countries and years)–30 291 (Ukraine, 2022)
Prevalence of chronic hepatitis C by country of birth	Polaris Observatory [36]	2020^d^	0.1% (several countries)–4.7% (Gabon)
Proportion of immigrants among active PWID with chronic hepatitis C	Prevalence surveys among PWID attending low-threshold health and social care services in Oslo, Bergen, and Trondheim [12, 13, 22, 26]	2007–2022^c^	10%
Treatment			
Number	Expert opinion and studies in Norway [37–39]	1990–1999	9 (1990)–228 (1999)
Number	Norwegian Prescribed Drug Registry	2004–2022	195 (2005)–2954 (2018)
Rate of treatment uptake among active PWID with chronic hepatitis C^e^	Registry-based study among PWID attending low-threshold health and social care services in Oslo [14]	2010–2019	0.34 per 100 (2010)–24.1 per 100 (2018)
Success (achieving a sustained virological response)	Studies in Norway [37–41]	1990–2011	30% (1990–1993)–70% (2000–2010)
Success (achieving a sustained virological response)	Data from the Norwegian Prescribed Drug Registry on the proportion of direct-acting antiviral^f^ treatment courses, when complete treatment was dispensed	2011–2022	72% (2011–2013)–90% (2017–2022)

Symbol — indicates stable value across all years.

Abbreviations: COVID-19, coronavirus disease 2019; NSP, needle and syringe programs; OST, opioid substitution therapy; PWID, people who inject drugs.

^a^Further details on the range of values used in the model are described in the manuscript and Supplementary Materials A. Values without a range were kept constant for all years.

^b^Excludes 2020. Estimates of the number of active PWID are based on the number of overdose deaths. An estimate for 2020 was not generated because the increase in overdose deaths that year is likely due to the COVID-19 pandemic and related closure of many low-threshold harm reduction facilities.

^c^Surveys were not conducted in all locations in all years. No surveys were conducted at any location in 2013, 2014, and 2019. For the element “Proportion of immigrants among active PWID with chronic hepatitis C,” we also did not have access to data from the survey in 2012.

^d^OST was first available in Norway in 1998.

^e^For treatments not allocated to active PWID, we assumed that the per-person annual probability that ex-PWID or immigrants would seek treatment was the same.

^f^Direct-acting antivirals includes medicines with the Anatomical Therapeutic Chemical codes J05AP02–J05AP57. A full description of these data is available in Supplementary Material A.

Below, we further describe the input data and their use in the baseline model. More comprehensive details on the model, input data, and baseline model fits are presented in Supplementary Material A. The model code and input data are available at https://github.com/folkehelseinstituttet/hepatitis_C_model/.

### People Who Inject Drugs

Estimates of the number of active PWID were calculated using the mortality multiplier method, and transmission rates between active PWID, ex-PWID temporarily ceased, and ex-PWID permanently ceased were based on literature, as previously described [29, 30]. We assumed an increasing annual mortality rate over time, as the population aged. For active PWID, we assumed an increased mortality rate (0.03 per person per year) due to overdose deaths [34].

For β, we assumed that surveys among PWID attending low-threshold health and social care services in different cities in Norway from 2002 to 2022 were representative of the prevalence among active PWID nationally. The average time spent in the compartment for acute infection was 6 months, and we assumed a spontaneous clearance rate of 26% [31]. Active PWID who had recovered could be reinfected at the same rate as hepatitis C naive active PWID. NSP and OST reduced the effective infection rate by a value informed by the point estimate in Platt et al [32] and changes in the coverage of these interventions over time. NSP coverage was assumed to increase from 0% in 1987 to 77%, 80%, 87%, and 92% in 2016–2019 [24], after which it was held constant. The number of OST patients has increased over time to >8300 in 2022 [21]. We assumed that 20% of OST patients per year overlapped with active PWID. Furthermore, as in previous modelling in Norway [28], we used a Gini coefficient to reduce the rate of infection over time to reflect the increasing geographical spread in the injecting epidemic and decreasing chance that 2 active injectors will meet.

To make projections for PWID until 2030, the model assumed the same infection rate, proportion seeking treatment, and rate of PWID debut as in 2022. Mortality rates and the Gini coefficient were projected along the same trend as in the past.

### Immigrants

Data from Statistics Norway informed the net annual immigration by country of birth [35]. As no data exist on the prevalence of chronic hepatitis C among immigrants to Norway, we assumed that this was the same as in the country of birth, using point estimates from the Polaris Observatory [36]. For countries without a national estimate, we used the Global Burden of Disease regional estimates [36]. We assumed that infection does not occur after arrival to Norway. However, to consider the overlap between PWID and immigrants, we assumed a gradual leakage of immigrants with chronic hepatitis C to active PWID, such that immigrants constituted 10% of active PWID with chronic hepatitis C in 2022. This assumption was guided by data from the prevalence surveys and the understanding that immigrants constitute a higher proportion of the population in Oslo than nationally.

### Treatment

The annual number of treatments and treatment success (achieving a sustained virological response) rate nationally were based on data from published studies [37–41] and the Norwegian Prescribed Drug Registry. For active PWID, we based the rate of treatment uptake on the registry-based study among active PWID diagnosed with hepatitis C who had attended low-threshold health and social care services in Oslo, rising to a constant of 22.1/100 person-years from 2019 [14]. For the remaining unallocated treatments, we assumed that the per-person annual probability that ex-PWID or immigrants would seek treatment was the same. This probability was varied each year by the model, so the total number of treatments matched the data. Treatment success increased gradually to 90% from 2017, and was not varied by risk group.

### Counterfactual Scenarios and Sensitivity Analyses

To investigate the effect of treatment scale-up, we ran models assuming no scale-up from 2013, before the introduction of interferon-free DAA. To investigate the robustness of our baseline model, we also conducted a wide range of sensitivity analyses, several of which are described in the “Results” section. A full summary of all sensitivity analyses is available in Supplementary Material A.

### Ethics

The model only utilized anonymized and aggregated input data. Ethical approval was not considered necessary.

## RESULTS

### Overall

**Figure 2.**
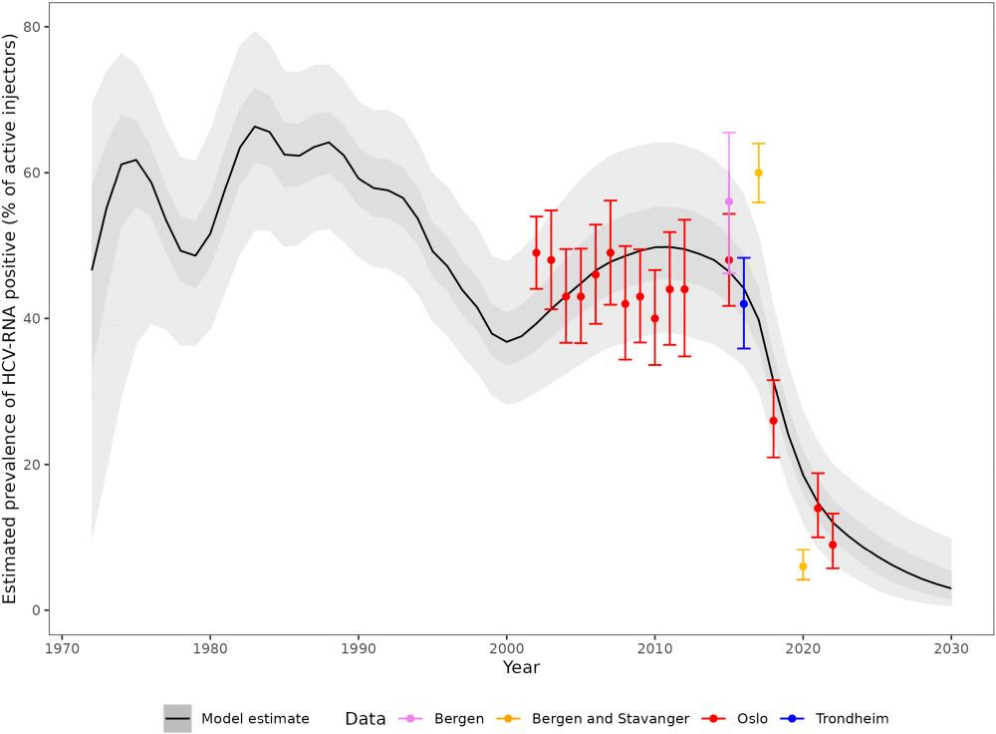
Model fit among active people who inject drugs to data on hepatitis C prevalence from surveys among people who inject drugs attending low-threshold health and social care services, 1972–2022. For the survey data, circles represent point estimates and error bars the 95% confidence intervals. For model estimates, the solid line is the median value, the darker shaded band is the interquartile credible interval, and the lighter shaded band is the 95% credible interval. Full results for the baseline model are available in Supplementary Material B. The setting and recruitment for these survey data are described in [12, 13, 22, 26]. For Oslo, the setting and method of recruitment have remained relatively unchanged since the first survey in 2002, aside from the addition of payment for participation in 2012. For Bergen alone, the setting and recruitment were similar to the study in Oslo [26]. For Bergen and Stavanger, the setting is described in [22], and the prevalence data are presented in [13]. The trend in model estimates prior to the first data point being available should be interpreted with caution.

**Figure 3.**
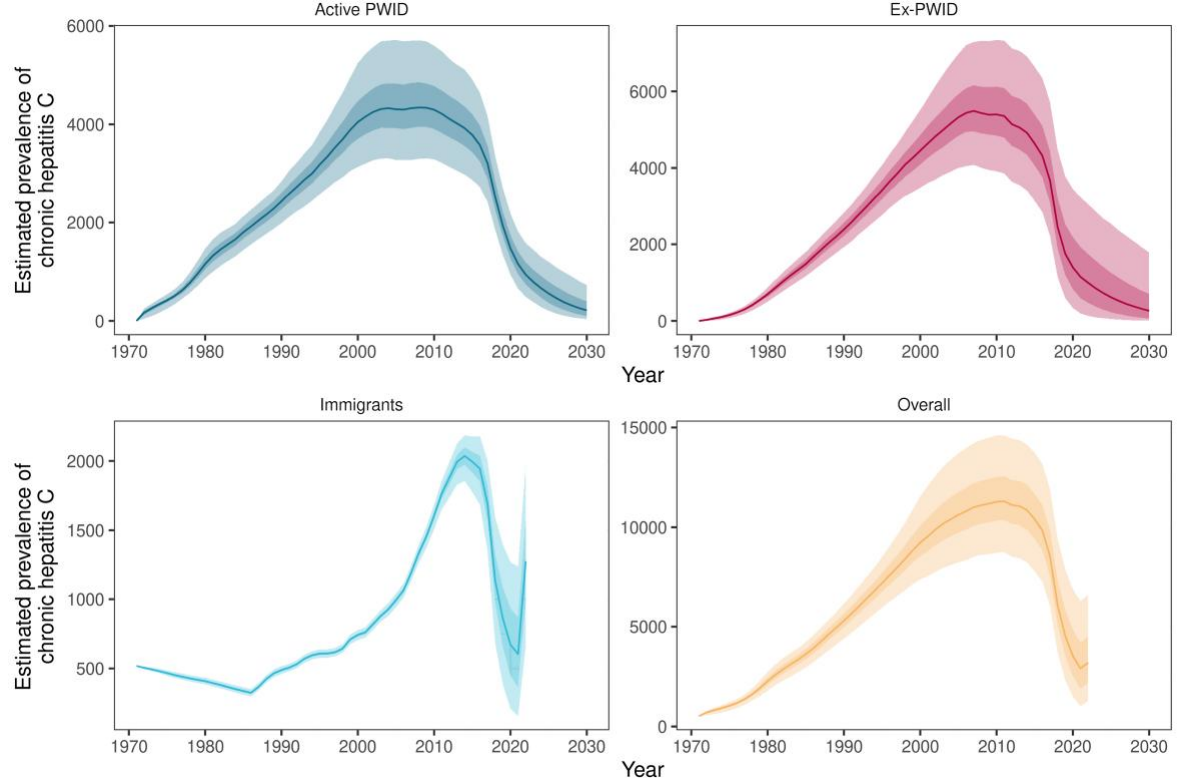
Estimated prevalence of chronic hepatitis C infection, by year and risk group, Norway, 1972–2030. The solid line is the median value, the darker shaded band is the interquartile credible interval, and the lighter shaded band is the 95% credible interval. Projections until 2030 are only presented for active and ex-PWID. For immigrants and the model including all risk groups, estimates are only modelled until the last year with available data on immigrants (2022). Full results for the baseline model are available in Supplementary Material B. Abbreviation: PWID, people who inject drugs.

The baseline model fit to prevalence data from surveys among PWID is presented in Figure 2. Other model fits to data are presented in Supplementary Material A. The model estimated an increasing prevalence of chronic hepatitis C among PWID and immigrants, until a peak of 11 306 (95% Crl, 8743–14 617) in 2011, after which it decreased to 3202 (95% Crl, 1273–6601) infected persons in 2022 (Figure 3 and Table 2). The per-person annual probability of seeking treatment for ex-PWID and immigrants reached 0.41 (95% Crl, 0.17–0.63) in 2018, before decreasing to 0.22 (95% Crl, 0.07–0.63) in 2022.

**Table 2.** Summary of Results for the Prevalence of Chronic Hepatitis C in the Baseline Model, and Selected Counterfactual Scenarios and Sensitivity Analyses, by Risk Group, Norway

Model	Peak Prevalence Year	Peak Prevalence (95% Crl)	Prevalence in 2015 (95% Crl)	Prevalence in 2022 (95% Crl)	Relative Change in Prevalence, 2022 Compared to 2015 (95% Crl)
Overall					
Baseline	2011	11 306 (8743–14 617)	10 395 (7786–13 743)	3202 (1273–6601)	0.31 (0.16–0.48)
NSP effect adjustment	2011	11 192 (8491–14 386)	10 437 (7694–13 674)	3485 (1348–6961)	0.33 (0.18–0.51)
OST effect adjustment	2011	11 323 (8614–14 533)	10 397 (7657–13 666)	3194 (1274–6545)	0.31 (0.17–0.48)
Ukraine prevalence adjustment	2011	11 393 (8741–14 591)	10 455 (7760–13 672)	2735 (913–6099)	0.26 (0.12–0.45)
COVID-19 adjustment	2011	11 321 (8786–14 531)	10 390 (7867–13 656)	3202 (1354–6598)	0.31 (0.17–0.48)
No treatment scale-up 1	2011	11 799 (8298–14 072)	11 217 (7555–13 525)	9770 (6153–12 099)	0.87 (0.81–0.89)
No treatment scale-up 2	2011	11 634 (8342–13 986)	11 210 (7617–13 490)	10 354 (6680–12 722)	0.92 (0.88–0.94)
Active PWID					
Baseline	2008	4342 (3297–5709)	3784 (2867–4914)	940 (486–1583)	0.25 (0.17–0.32)
NSP effect adjustment	2008	4330 (3235–5616)	3892 (2918–4986)	1034 (546–1731)	0.27 (0.19–0.35)
OST effect adjustment	2008	4345 (3255–5638)	3780 (2839–4891)	932 (483–1578)	0.25 (0.17–0.32)
Ukraine prevalence adjustment	2008	4386 (3320–5699)	3808 (2880–4908)	920 (463–1557)	0.24 (0.16–0.32)
COVID-19 adjustment	2008	4338 (3330–5699)	3764 (2869–4886)	1000 (549–1680)	0.27 (0.19–0.34)
No treatment scale-up 1	2008	4545 (3087–5462)	4011 (2709–4858)	3100 (1939–3854)	0.77 (0.72–0.79)
No treatment scale-up 2	2008	4481 (3109–5519)	3992 (2710–4853)	3217 (2061–3957)	0.81 (0.76–0.82)
Ex-PWID					
Baseline	2007	5490 (4077–7289)	4634 (3129–6643)	993 (129–3052)	0.21 (0.04–0.46)
NSP effect adjustment	2007	5240 (3827–6963)	4574 (2981–6512)	1113 (137–3203)	0.24 (0.05–0.49)
OST effect adjustment	2007	5454 (4015–7218)	4631 (3062–6610)	987 (123–2993)	0.21 (0.04–0.45)
Ukraine prevalence adjustment	2007	5552 (4123–7302)	4702 (3126–6652)	968 (120–3025)	0.21 (0.04–0.45)
COVID-19 adjustment	2007	5482 (4119–7231)	4635 (3173–6564)	954 (137–2965)	0.21 (0.04–0.45)
No treatment scale-up 1	2007	5739 (3878–6921)	5105 (2995–6443)	4225 (2158–5647)	0.83 (0.72–0.88)
No treatment scale-up 2	2007	5680 (3879–6977)	5106 (3027–6510)	4547 (2436–5991)	0.89 (0.80–0.92)
Immigrants					
Baseline	2014	2036 (1854–2186)	1995 (1776–2176)	1278 (657–1979)	0.64 (0.37–0.91)
NSP effect adjustment	2014	2023 (1826–2180)	1979 (1750–2174)	1331 (661–2003)	0.67 (0.38–0.92)
OST effect adjustment	2014	2034 (1851–2188)	1988 (1768–2185)	1272 (654–1974)	0.64 (0.37–0.90)
Ukraine prevalence adjustment	2014	1994 (1820–2148)	1949 (1742–2134)	835 (330–1503)	0.43 (0.19–0.70)
COVID-19 adjustment	2014	2036 (1864–2188)	1991 (1787–2180)	1239 (661–1925)	0.62 (0.37–0.88)
No treatment scale-up 1	2022	2466 (2062–2679)	2089 (1836–2232)	2466 (2062–2679)	1.18 (1.12–1.20)
No treatment scale-up 2	2022	2604 (2192–2786)	2114 (1857–2247)	2604 (2192–2786)	1.23 (1.18–1.24)

NSP effect adjustment: NSP reduces the effective infection rate by 20% (56% in baseline). OST effect adjustment: OST reduces the effective infection rate by 37% (50% in baseline). Ukraine prevalence adjustment: Over 30 000 immigrants born in Ukraine arrived in Norway in 2022. In this analysis we assume a 1% prevalence of chronic hepatitis C among immigrants from Ukraine (3.1% in baseline). This is because, while the prevalence of hepatitis C in Ukraine has been estimated to be higher among older men [42], 63% of immigrants from Ukraine to Norway in 2022 were women, and among settled immigrants from Ukraine two-thirds are <40 years old. COVID-19 adjustment: While the impact of the COVID-19 pandemic on the epidemiology on hepatitis C among PWID in Norway is uncertain, some low-threshold testing services and harm reduction services closed for extended periods, and the number of needles and syringes distributed decreased [13]. We therefore assume that NSP coverage decreased to 70% (92% in baseline) and a 25% reduction in treatment uptake among active PWID (from 22.1/100 person-years in baseline) in 2020 and 2021. No treatment scale-up 1: Assume same rate of treatment uptake from 2013 onwards. Model also not fitted to HCV RNA prevalence data from surveys among PWID attending low-threshold health and social care services from 2013 onwards. No treatment scale-up 2: Assume same rate of treatment uptake and treatment success from 2013 onwards. Model also not fitted to HCV RNA prevalence data from surveys among PWID attending low-threshold health and social care services from 2013 onwards. A comparable table with results for all counterfactual scenarios and sensitivity analyses is presented in Supplementary Material A. Full results for the baseline model are available in Supplementary Material B.

Abbreviations: COVID-19, coronavirus disease 2019; CrI, credible interval; HCV, hepatitis C virus; NSP, needle and syringe programs; OST, opioid substitution therapy; PWID, people who inject drugs.

### People Who Inject Drugs

**Figure 4.**
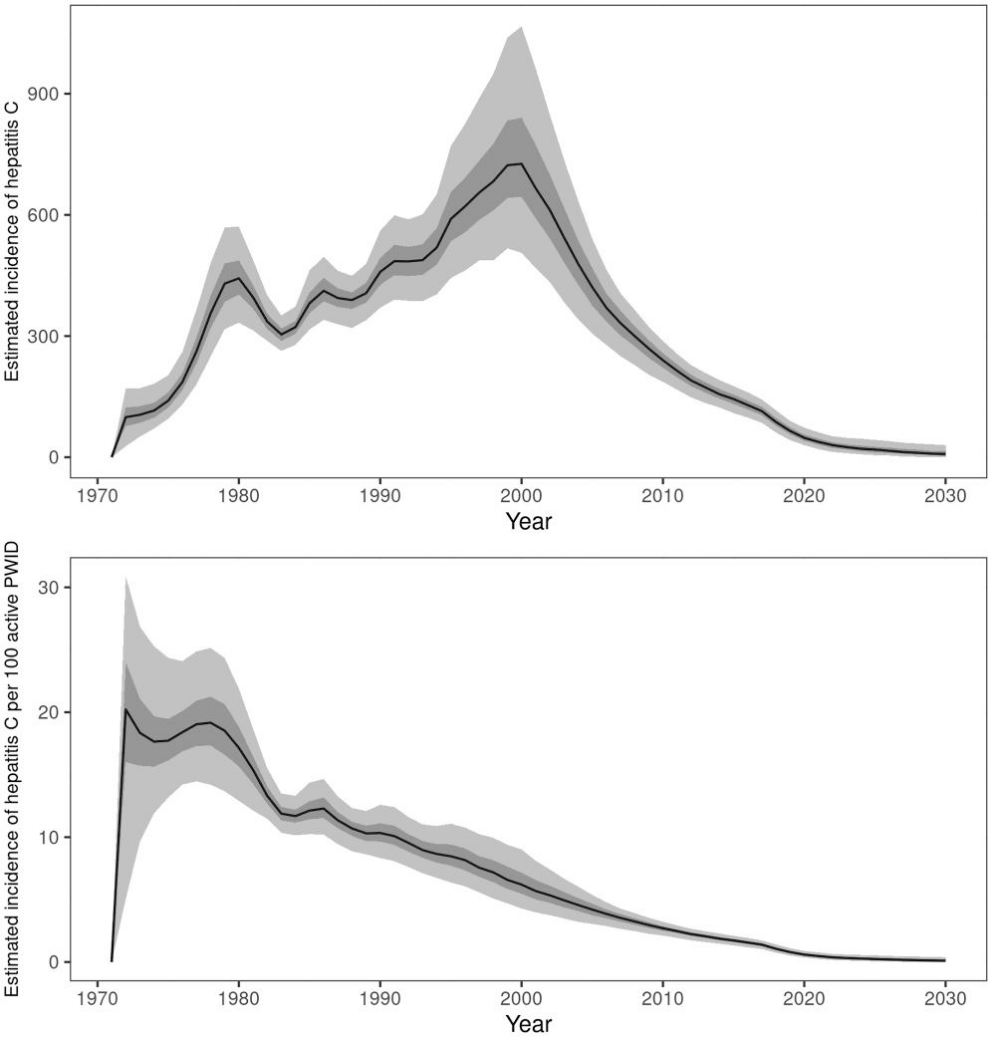
Estimated incidence of hepatitis C infection (number and per 100) among active PWID, by year, Norway, 1972–2030. The solid line is the median value, the darker shaded band is the interquartile credible interval, and the lighter shaded band is the 95% credible interval. Full results for the baseline model are available in Supplementary Material B. Abbreviation: PWID, people who inject drugs.

Among active PWID, incidence increased to a peak of 726 (95% Crl, 506–1067) new infections in 2000, before steadily decreasing to 30 (95% Crl, 13–52), or 0.37/100 active PWID (95% Crl, 0.17–0.65), in 2022. The estimated median incidence and 95% Crl were under <2/100 PWID from 2016 (1.56; 95% Crl, 1.18–1.93). There was an estimated 79% (95% Crl, 70%–88%) relative decrease in incidence in 2022, compared to 2015. This relative decrease was projected to continue to 94% (95% Crl, 83%–100%) by 2030 (Figure 4 and Table 3). Prevalence among active PWID peaked in 2008 (4342; 95% Crl, 3297–5709) and decreased to 940 (95% CrI, 486–1583) infected persons in 2022, with a comparable relative decrease, compared to 2015, as for incidence. This equated to an estimated prevalence of 11.8% (95% Crl, 6.2%–19.6%) among active PWID in 2022 (Figure 3 and Table 2). The median estimated prevalence among active PWID was predicted to first drop under 5% in 2028 (4.2%; 95% Crl, 1.0%–11.3%, or 316 infected persons; 95% Crl, 72–908). Among ex-PWID, the model followed a similar trend (Figure 3 and Table 2), with an estimated 80% (95% CrI, 60%–96%) decrease in prevalence in 2022 compared to 2015. The projected prevalence in 2030 was 378 (95% Crl, 16–2078).

**Table 3.** Summary of Results for the Incidence of Hepatitis C Among Active PWID in the Baseline Model, and Selected Counterfactual Scenarios and Sensitivity Analyses, Norway

Model	Peak Incidence Year	Peak Incidence (95% Crl)	Incidence in 2015 (95% Crl)	Incidence Per 100 in 2015 (95% Crl)	Incidence in 2022 (95% Crl)	Incidence Per 100 in 2022 (95% Crl)	Relative Change, 2022 Compared to 2015 (95% Crl)	Incidence in 2030 (95% Crl)	Incidence Per 100 in 2030 (95% Crl)	Relative Change, 2030 Compared to 2015 (95% Crl)
Baseline	2000	726 (506–1067)	144 (109–176)	1.72 (1.33–2.10)	30 (13–52)	0.37 (0.17–0.65)	0.21 (0.12–0.30)	8 (0–52)	0.11 (0.00–0.38)	0.06 (0.00–0.17)
NSP effect adjustment	2000	728 (496–1030)	182 (145–217)	2.18 (1.76–2.59)	47 (22–77)	0.59 (0.28–0.95)	0.26 (0.15–0.35)	15 (1–77)	0.20 (0.02–0.62)	0.08 (0.01–0.23)
OST effect adjustment	2000	723 (490–1041)	146 (112–178)	1.75 (1.34–2.11)	30 (13–52)	0.38 (0.17–0.65)	0.21 (0.12–0.29)	8 (0–52)	0.11 (0.00–0.39)	0.05 (0.00–0.17)
Ukraine prevalence adjustment	2000	739 (502–1071)	145 (113–176)	1.74 (1.36–2.10)	30 (14–53)	0.38 (0.18–0.66)	0.21 (0.12–0.30)	6 (0–53)	0.09 (0.00–0.34)	0.04 (0.00–0.15)
COVID-19 adjustment	2000	727 (504–1049)	143 (111–172)	1.71 (1.33–2.06)	33 (16–57)	0.41 (0.20–0.70)	0.23 (0.14–0.33)	8 (0–57)	0.10 (0.00–0.37)	0.06 (0.00–0.17)
No treatment scale-up 1	2000	769 (441–1008)	148 (111–182)	1.78 (1.31–2.16)	76 (48–98)	0.97 (0.61–1.25)	0.51 (0.43–0.54)	58 (33–98)	0.82 (0.47–1.17)	0.39 (0.29–0.47)
No treatment scale-up 2	2000	754 (457–997)	145 (107–179)	1.72 (1.27–2.12)	77 (53–100)	0.98 (0.67–1.27)	0.53 (0.50–0.56)	62 (38–100)	0.88 (0.53–1.19)	0.43 (0.35–0.48)

NSP effect adjustment: NSP reduces the effective infection rate by 20% (56% in baseline). OST effect adjustment: OST reduces the effective infection rate by 37% (50% in baseline). Ukraine prevalence adjustment: Over 30 000 immigrants born in Ukraine arrived in Norway in 2022. In this analysis we assume a 1% prevalence of chronic hepatitis C among immigrants from Ukraine (3.1% in baseline). This is because, while the prevalence of hepatitis C in Ukraine has been estimated to be higher among older men [42], 63% of immigrants from Ukraine to Norway in 2022 were women, and among settled immigrants from Ukraine two-thirds are <40 years old. COVID-19 adjustment: While the impact of the COVID-19 pandemic on the epidemiology on hepatitis C among PWID in Norway is uncertain, some low-threshold testing services and harm reduction services closed for extended periods, and the number of needles and syringes distributed decreased [13]. We therefore assume that NSP coverage decreased to 70% (92% in baseline) and a 25% reduction in treatment uptake among active PWID (from 22.1/100 person-years in baseline) in 2020 and 2021. No treatment scale-up 1: Assume same rate of treatment uptake from 2013 onwards. Model also not fitted to HCV RNA prevalence data from surveys among PWID attending low-threshold health and social care services from 2013 onwards. No treatment scale-up 2: Assume same rate of treatment uptake and treatment success from 2013 onwards. Model also not fitted to HCV RNA prevalence data from surveys among PWID attending low-threshold health and social care services from 2013 onwards. A comparable table with results for all counterfactual scenarios and sensitivity analyses is presented in Supplementary Material A. Full results for the baseline model are available in Supplementary Material B.

Abbreviations: COVID-19, coronavirus disease 2019; CrI, credible interval; HCV, hepatitis C virus; NSP, needle and syringe program; OST, opioid substitution therapy; PWID, people who inject drugs.

### Immigrants

Prevalence among immigrants peaked in 2014 and decreased to 604 (95% Crl, 156–1238) infected persons in 2021. Prevalence increased again in 2022 (Figure 3 and Table 2). The estimated net immigration of people with chronic hepatitis C to Norway by country and year is presented in Supplementary Material A.

### Counterfactual Scenarios and Sensitivity Analyses

Assuming no further scale-up in the number of treatments from 2013, the estimated incidence among active PWID in 2022 was 76 new infections (95% Crl, 48–98), or 0.97/100 active PWID (95% Crl, 0.61–1.25). The relative decrease compared to 2015 was 49% (95% Crl, 46%–57%; Table 3). Under this scenario, the estimated prevalence among active PWID in 2022 was 39% (95% Crl, 25%–49%) and was not predicted to drop under 32% (95% Crl, 18%–42%) by 2030, a prevalence of 2315 infected persons (95% Crl, 2024–2985). Prevalence in 2022 increased notably among all groups, compared to the baseline model (Table 2). Additionally, assuming the same rate of treatment success from 2013 onwards resulted in further increases in the estimated incidence and prevalence (Table 2).

Results were not noticeably affected by the sensitivity analyses presented in Table 2 and Table 3. Small variations, for example, were a slightly higher incidence among active PWID, when assuming a lower reduction in the risk of HCV infection by NSP. Results in all other sensitivity analyses presented in Supplementary Material A, for example assuming larger or smaller PWID populations, different rates of spontaneous clearance of acute infection, or a lower treatment rate among active PWID, were generally consistent with the baseline model or varied as expected. In all sensitivity analyses, the incidence in 2022 was below the WHO absolute target, including in a more pessimistic scenario assuming a lower reduction in the risk of HCV infection by NSP and OST, lower overall number of treatments, and lower treatment uptake among active PWID.

## DISCUSSION

Our model estimated that the incidence of hepatitis C among active PWID in Norway in 2022 was 30 (95% Crl, 13–52) new infections, or 0.37/100 (95% Crl, 0.17–0.65). With an incidence of deaths due to HCV infection <1/100 000 [13], and considering that the epidemic is concentrated in PWID, this suggests that Norway has achieved both WHO absolute impact targets for the elimination of hepatitis C as a public health threat. We would have had to underestimate incidence several fold to breach the incidence target, which gives confidence in this conclusion. Also, the relative decrease in incidence among active PWID in 2022 was on the cusp of the global relative target (≥80%, compared to 2015) [25], and model prognoses suggest that Norway is on track to reach the national incidence target (≥90% decrease, compared to 2015) [16]. This is supported by expected similar relative decreases in prevalence [27]. Interestingly, in neighboring Sweden, modelling estimated that a 90% decrease in incidence could be achieved if 90% of PWID were engaged in NSP by 2023, and 21% of HCV-infected PWID engaged in harm reduction services were treated annually [43], approximately the level that data suggest Norway is at [14, 24].

Few countries are on track to reach the elimination targets [7]. According to the European Monitoring Centre for Drugs and Drug Addiction, among European Union countries plus Türkiye and Norway, Norway is the only country with data showing a significant reduction in HCV RNA prevalence among PWID over time [44]. Our results provide an example of what can be achieved in a setting with a concentrated epidemic, range of low-threshold and outreach services, high coverage of harm reduction services, simplified and integrated treatment pathways, and high rate of treatment uptake and completion. Indeed, our model suggests that Norway may have been under the WHO absolute incidence target among PWID from 2016, highlighting the benefit of high NSP and OST coverage in preventing HCV transmission. In contrast to counterfactual scenarios, our model also demonstrates how critical recent treatment scale-up has been to further reduce the incidence and prevalence of hepatitis C. Other modelling studies have also shown the real-world impact of treatment scale-up on progress towards hepatitis C elimination [45, 46]. In 2023, some European Union countries still imposed restrictions on DAA access [44].

However, the estimated prevalence of chronic infection across the 3 risk groups in 2022 was around 3000 active infections, with likely ongoing transmission among active PWID in Norway until 2030. These estimates also do not cover other risk groups, understood not to be important drivers of hepatitis C transmission nor carry a notable disease burden in Norway, but still considered at risk of infection and recommended for testing [13, 17]. As also highlighted by others [43, 45, 46], continued momentum and vigilance is essential as elimination approaches. To this end, the necessary renewal of the Norwegian national strategy for viral hepatitis [13, 16] provides a timely opportunity to review the focus, approach, and goals. An example for potential further service improvement in Norway is that still some active PWID live in municipalities without NSP [24]. Also, the recent roll out of home sampling in England [47] and Ireland [48] shows how testing strategies may be further enhanced.

Our model was informed by a comprehensive range of real-world data sources that represent the best available knowledge on the hepatitis C epidemic and related parameters for the modelled risk groups in Norway. The wide range of sensitivity analyses did not challenge the conclusions and provide useful information on how much estimates vary under a different set of assumptions. Similar to an earlier Markov natural history model among PWID in Norway [28], we found that the incidence of hepatitis C among active PWID rose to around 500 new infections per year around 1990, and then continued to a peak of around 800 new infections around the year 2000. Prevalence peaked among active PWID at around 4500 infected persons in 2002–2003 in both models. However, our incidence curve falls more than twice as fast from the turn of the century and we thereafter consistently find a higher prevalence and lower incidence among active PWID. While other differences in the modelling approach must also be considered, this may be due to input data on treatments, as the flat treatment rate in the earlier model appears to give an unrealistically high number of treatments among active PWID, compared to ours based on observed data. Both models used data from the prevalence surveys in Oslo to inform the underlying infection rate, so a higher treatment rate may explain why the earlier model estimated higher incidence, but lower prevalence.

However, while our results are encouraging, some key limitations must be considered. We assumed that the HCV prevalence data from surveys among PWID attending low-threshold health and social care services (many of which were conducted in Oslo) and study on treatment uptake among active PWID in Oslo were representative of active PWID nationally. These assumptions may be reasonable, considering the relative consistency in survey estimates between locations for overlapping years, robustness of incidence estimates when assuming both a lower and higher treatment rate among active PWID, nationally high coverage of harm reduction services, and widespread national and local information campaigns by state and user organizations [13]. Furthermore, different outreach services in Oslo [18] and outside large cities [19], that were not included in the model inputs, have also reported decreasing HCV prevalence among their target groups to around 10% of those tested [13] (personal communication, Ronny Bjørnestad, proLAR Nett). Similar prevalence estimates have also been reported among patients on OST (approximately 85% of 8300 OST patients in 2022 had reported hepatitis C status, of which around 7.5% had an active infection) [21], while among prisoners (who largely overlap with PWID, and are therefore somewhat covered by our model) a 2018–2019 study in 6 prisons found an HCV RNA prevalence of 9.3% [13]. Nonetheless, the accuracy of our assumptions should be validated in future biobehavioral surveys among active PWID, including from a wider range of geographical areas around Norway. Ideally, such surveys will also allow some empirical validation of the modelled incidence estimates.

Also, given fluctuating patterns of immigration and the importance of immigrants to the hepatitis C epidemic in Norway, it was of interest to include immigrants in this model. We assumed that the prevalence of chronic hepatitis C among immigrants mirrored the estimated prevalence in the country of birth. Studies from England and the Netherlands have questioned the validity of this assumption [49, 50]. A similar example in our study regards the estimated prevalence among immigrants in 2022, given the composition of immigrants from Ukraine. In our model, false assumptions on the prevalence among immigrants have a knock-on effect on ex-PWID, as we assumed the same per-person annual probability of seeking treatment. While our results may provide a guiding range for the prevalence of chronic hepatitis C among immigrants in Norway, the lack of empirical data is a priority gap to fill.

In this study, we generated bespoke estimates of the incidence and prevalence of hepatitis C among PWID and immigrants in Norway. Results indicate that Norway has achieved the WHO absolute incidence target for the elimination of hepatitis C as a public health threat and provide a new baseline to guide efforts to further reduce the transmission and burden of hepatitis C. Model assumptions and outputs should be validated against empirical data from future studies.

## Supplementary Data

Supplementary materials are available at *The Journal of Infectious Diseases* online (http://jid.oxfordjournals.org/). Supplementary materials consist of data provided by the author that are published to benefit the reader. The posted materials are not copyedited. The contents of all supplementary data are the sole responsibility of the authors. Questions or messages regarding errors should be addressed to the author.

## Supplementary Material

jiae147_Supplementary_Data

## Notes

***Author contributions*.** R. W. and H. K. conceived the idea for the study and established the registry-linkage study that provided some input data on hepatitis C treatments. R. W. and J. M. designed the model. R. W. collated all input data for use in the model. J. M. developed the model code and ran all model analyses. R. W. and J. M. drafted the manuscript. All authors contributed to the revision of the manuscript and approved the final version for submission.

***Acknowledgments*.** We thank Jasmina Burdzovic and Ellen Amundsen (The Norwegian Institute of Public Health), Lars Fadnes and Jørn Henrik Vold (University of Bergen), Eirik Opheim and Kjersti Ulstein (Municipality of Oslo), Thomas Clausen (Center for Addiction Research), and Aase-Britt Holmboe (The Norwegian Hospital Procurement Trust) for providing input data used in the model. We also thank Sasikiran Kandula (The Norwegian Institute of Public Health) for his input to model design.

***Financial support*.** No financial support was received for this work.
