# Supplementary material for: Monitoring Progress Towards the Elimination of Hepatitis C as a Public Health Threat in Norway: A Modelling Study Among People Who Inject Drugs and Immigrants
Source: J Infect Dis. 2024 Mar 27;230(3):e700–11. doi: 10.1093/infdis/jiae147 (PMC11420790; doi:10.1093/infdis/jiae147)
Supplement: jiae147_Supplementary_Data [file jiae147_supplementary_data.zip › supplement_A_model_hepatitis C_Norway.docx]

**Supplement A to the article *Monitoring progress towards the elimination of hepatitis C as a public health threat in Norway: a modelling study among people who inject drugs and immigrants*.**

**Contents**

[Details of the model 2](#_Toc160712409)

[Model framework 2](#_Toc160712410)

[Model fit 4](#_Toc160712411)

[Estimates of the number of PWID 8](#_Toc160712412)

[Estimated reduction in the effective infection rate due to NSP and OST 9](#_Toc160712413)

[NSP coverage 9](#_Toc160712414)

[Number of patients on OST 10](#_Toc160712415)

[Estimated reduction in the effective infection rate 10](#_Toc160712416)

[Gini coefficient 11](#_Toc160712417)

[Model fit to data on HCV antibody prevalence among PWID in Oslo. 12](#_Toc160712418)

[Estimated net immigration of people with chronic hepatitis C to Norway 13](#_Toc160712419)

[Proportion of PWID in prevalence surveys born outside Norway 14](#_Toc160712420)

[Treatment input data 15](#_Toc160712421)

[Number of treatments 16](#_Toc160712422)

[Treatment success 20](#_Toc160712423)

[Counterfactual scenarios and sensitivity analyses 21](#_Toc160712424)

[References 32](#_Toc160712425)

# Details of the model

The model is a stochastic compartmental model implemented in R using the odin/dust/mcstate framework [1, 2]. See the main manuscript, section ‘Materials and methods’, for a summary of the model, Figure 1 in the main manuscript for a schematic overview and Table 1 in the main manuscript for a summary of all input data. Below, we give further details on the model framework and fit. In subsequent sections in this supplement, further detail on input data, model fit to data and sensitivity analyses are also presented. The model R code and input data are available at <https://github.com/folkehelseinstituttet/hepatitis_C_model/>.

## Model framework

The model divides the study population into compartments. Time is discretised, with a resolution of ¼-year time-steps. At each time-step, the number of persons transitioning between compartments is calculated using a binomial probability:

$$n_{A\to B} = binom\left( N_{A}^{i-1}; p_{A\to B}^{individ} \right)$$

$n_{A\to B}$ is the number of persons transitioning; $N_{A}^{i-1}$ is the number of persons in compartment A at the previous time-step; and $p_{A\to B}^{individ}$ is the per-person probability of transitioning from compartment A to compartment B. If several different transition paths are possible from one source compartment, we take this into account so that we do not deplete the source compartment of more individuals than its total population. The per-person transition probability is calculated from the corresponding transition rate by the formula:

$$p_{A\to B} = 1 - e^{-rate_{A\to B}\cdot\Delta t}$$

This formula follows from assuming that transitions are exponentially distributed and integrating the cumulative transition probability over the time interval $\Delta t$.

The model considers people who inject drugs (PWID) and immigrants. PWID are considered to belong to one of three groups: active, ex-PWID who have temporarily ceased injecting and ex-PWID who have permanently ceased injecting. Inside each group, we keep track of an individual’s infection status by considering them to be either hepatitis C virus (HCV) naïve (never infected, susceptible); acutely infected; chronically infected or recovered (through either spontaneous clearance or treatment). When a person changes their PWID status (e.g. active to ex-PWID who have temporarily ceased injecting), they keep their epidemiological status and will keep transitioning between compartments, e.g. from acute to chronic infection. Transmission of HCV infection between persons takes place only among active PWID. Transmission is estimated by calculating a force of infection (λ), which is the per-person probability of acquiring infection, given by

$$\lambda= \left( 1 - e^{-\beta\cdot\frac{AA+AC}{AN+AA+AC+AR}\cdot\Delta t\cdot g} \right)\cdot r$$

where $\beta$is the underlying infection rate (constant in time), $g$is the Gini coefficient of geographic dispersal, and $r$is the reduction in transmission risk due to needle and syringe programmes (NSP) and opioid substitution therapy (OST). For AN, AA, AC and AR, the first letter refers to active injectors, and the second letter (N, A, C and R) to the state of infection (naïve, acutely infected, chronically infected or recovered).

An acutely infected person spends an average of 6 months in this compartment and will either spontaneously clear their infection (probability 26%) or go on to become chronically infected. Once chronic, we assume that the only path to recovery is through treatment. The number of treatments are governed by a rate parameter. For active PWID this is estimated from data and input to the model, while for ex-PWID and immigrants the rate is estimated by a random-walking rate parameter fitted by a particle filter such that the total number of yearly treatments match data. We assume that recovered PWID may be reinfected with no reduced risk, however we want to keep track of them rather than move them back to the initial compartment in order to estimate antibody levels.

All compartments have a mortality rate, that increases over time to take into account the increasing mortality in an ageing population of PWID. The mortality rate is based on [3], which estimates the mean age of the PWID cohort over time., which estimates a mean age over time of the PWID cohort. We use this mean age to calculate an age-averaged mortality rate from Norwegian mortality data by convoluting the mean age using a Gaussian distribution to take into account the distribution of individuals’ age around the mean [4]. In the baseline, this Gaussian has a standard deviation of five years. Additionally, active PWID have an increased mortality rate of 0.03 per person per year to account for overdose deaths [5].

For immigrants, we assume that no transmission takes place within Norway. The immigrants instead enter in the model directly as chronically infected, assumed to have been (chronically) infected in their country of birth. The net (i.e. taking into account emigration and death) yearly immigration of people with chronic hepatitis C to Norway is estimated from data. We consider the overlap of the immigrant and PWID populations by including a rate of injecting debut for immigrants, whereby they are moved to the compartment for chronically infected active injectors.

## Model fit

The model is fitted to data using a particle Markov Chain Monte Caro (pMCMC) algorithm, by the pmcmc method in mcstate. We used a statistical particle filter with 10,000 particles. The total log-likelihood function used for the fit is

$$LL = LL_{active}+LL_{ex wr}+LL_{ex wnr}+LL_{HCV prev}+LL_{treat}$$

where $LL_{active}+LL_{ex wr}+LL_{ex wnr}$ are Poissonian log-likelihood terms comparing the total population each year of active, ex-PWID who have temporarily ceased injecting (will relapse) and ex-PWID who have permanently ceased injecting (will not relapse), respectively, to data; $LL_{HCV prev}$ is a binomial log-likelihood term comparing the fraction of PWID who are hepatitis C positive to data from prevalence surveys for the years when that is available, and $LL_{treat}$ is a Poissonian log-likelihood term comparing the total number of treated individuals in the model to data. Weakly informative truncated Gaussian priors are used for the three parameters inferred by the MCMC:

$$\beta\sim N\left( \mu=1.36, \sigma=5 \right), \beta\in\left[ 0.001, 10 \right]$$

$$\text{rate\_debut\_ini}\sim N\left( \mu=300, \sigma=600 \right), \text{rate\_debut\_ini}\in\left[ 0.01, \infty\right.]$$

$$\text{AA\_ini}\sim N\left( \mu=200, \sigma=300 \right), \text{AA\_ini}\in\left[ 0, 378 \right]$$

Two parameters are allowed to vary by random walk at each time-step. These are the rate of new active PWID and the per-person annual rate of seeking treatment for ex-PWID and immigrants (assumed to be the same for each group). For the former, the new proposed value at each time-step is determined by a multiplicative Gaussian random variable as

$$\text{rate\_debut}_{i+1} = \text{rate\_debut}_{i}\cdot a, a\sim N\left( \mu=1,\sigma=0.1 \right)$$

For the latter, rate_treatment, we constrain it to lie in the interval 0 – 2 (treatments per person per year) in order to help the particle filter avoid unrealistically high treatment rates. This is done by transforming the rate by a logit function before doing the random walk, and then backtransforming:

$$\text{rate\_treatment\_unbounded}_{i}=\log\left( \frac{\text{ }\text{rate\_treatment\_bounded}_{\text{i}}}{2-\text{rate\_treatment\_bounded}_{i}} \right))$$

$$\text{rate\_treatment\_unbounded}_{i+1}=\text{rate\_treatment\_unbounded}_{\text{i}}\cdot a, a\sim N\left( \mu=1,\sigma=0.35 \right)$$

$$\text{rate\_treatment\_bounded}_{i+1}=\frac{2}{1+\exp\left( -\text{rate\_treatment\_unbounded}_{\text{i+1}} \right)}$$

The pMCMC algorithm is run in two separate chains, each with 2,000 iterations of burn-in and then 5,000 iterations to sample from the posterior distribution. We use a thinning interval of five to select 1,000 samples from each chain. We verify that the chains are well-mixing and that the sampling has converged to the posterior by calculating the Gelman-Rubin convergence diagnostic, which is of the order 1.01 for all parameters. We also inspect the traceplot, shown in Figure 1 below. Figure 2 shows the pairwise scatterplot and marginal distributions of the parameters in the posterior distribution.

The traceplot reveals that some steps obtain a much lower likelihood value than their neighbours. This occurs because of the particle filter, since we use an option in *mcstate* to rerun every 100^th^ step for both the proposed and the accepted parameter value, in order to help “unstick” the chains. As a sensitivity analysis (nr 14c) we have inspected the effect of removing posterior samples with a likelihood value lower than –2500. Out of 2000 samples, this removes 165. This results in slightly smaller credible intervals on parameters, but no change in the point estimates, indicating that the resampling of parameter values does not lead to bias in the posterior estimation.


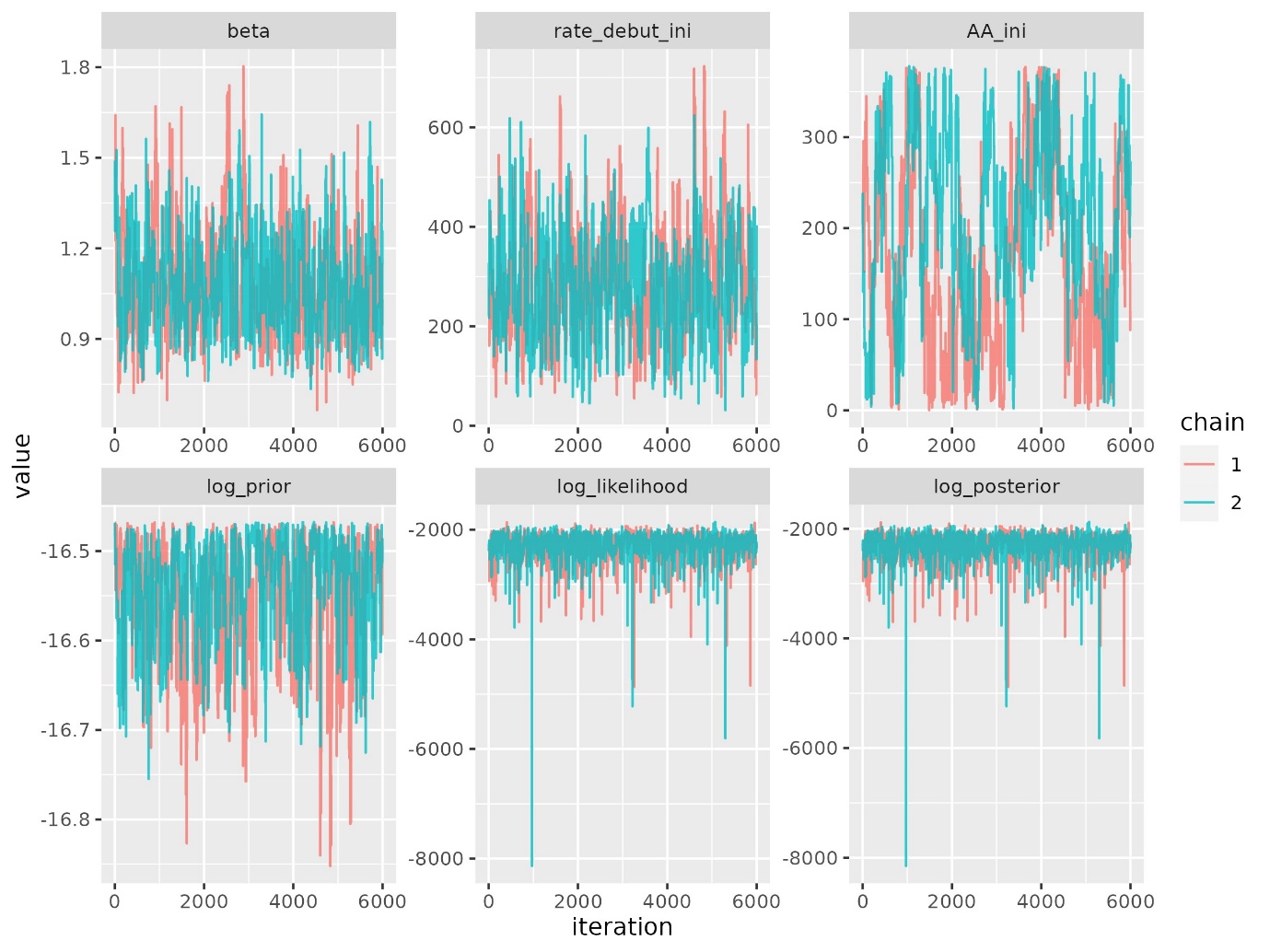


*Figure 1. Plot of the MCMC traces from the fit of the baseline model. The panels show the traces for each of the three variables under inference, for each of the two MCMC chains, as well as the logarithm of the prior, posterior and likelihood of the Bayesian statistical model.*

beta: underlying infection rate (constant in time). rate_debut_ini: rate of debut of new active injectors at the first time-step. AA_ini: number of PWID acutely infected at the first time-step.


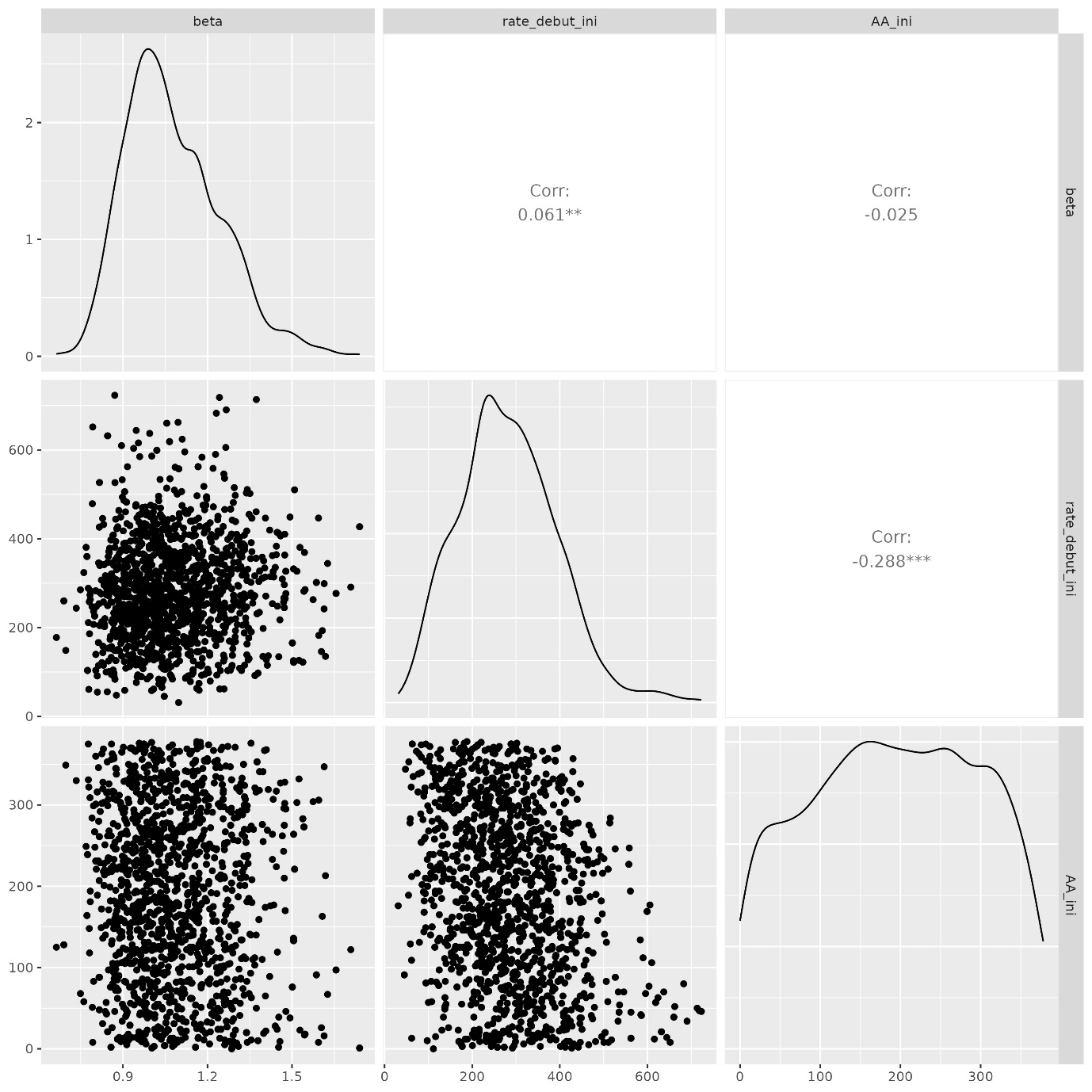


*Figure 2. Correlogram showing the pairwise distribution of the posterior samples of the baseline model.*

beta: underlying infection rate (constant in time). rate_debut_ini: rate of debut of new active injectors at the first time-step. AA_ini: number of PWID acutely infected at the first time-step.

# Estimates of the number of PWID

For PWID, we split the population into three groups; 1) active PWID, 2) ex-PWID who have temporarily ceased injecting and 3) ex-PWID who have permanently ceased injecting. Estimates of the size of each of these three groups came from The Norwegian Institute of Public Health, Department of Alcohol, Tobacco and Drugs. Estimates for active PWID are calculated using the Mortality Multiplier Method, and transmission rates between groups are based on literature, as described previously [6, 7]. The numerical values used for the rate of temporary cessation, permanent cessation and relapse, are 0.15/person-years, 0.025/person-years and 0.15/person-years, respectively, as described in [6]. Estimates were available from 1973 – 2021. For active PWID, we also used available credible intervals for these estimates from 2004 – 2021 to inform sensitivity analyses. We also did not include an estimate for 2020, as the increase in overdose deaths that year [5] is likely due to the COVID-19 pandemic and related closure of many low threshold harm reduction facilities. The model fit to these estimates is presented in Figure 3. It is assumed that the estimated peak in active PWID around the year 2000 is not real, so the model assumes that the population is constant at the level in 1999 from 2000 – 2002 inclusive.


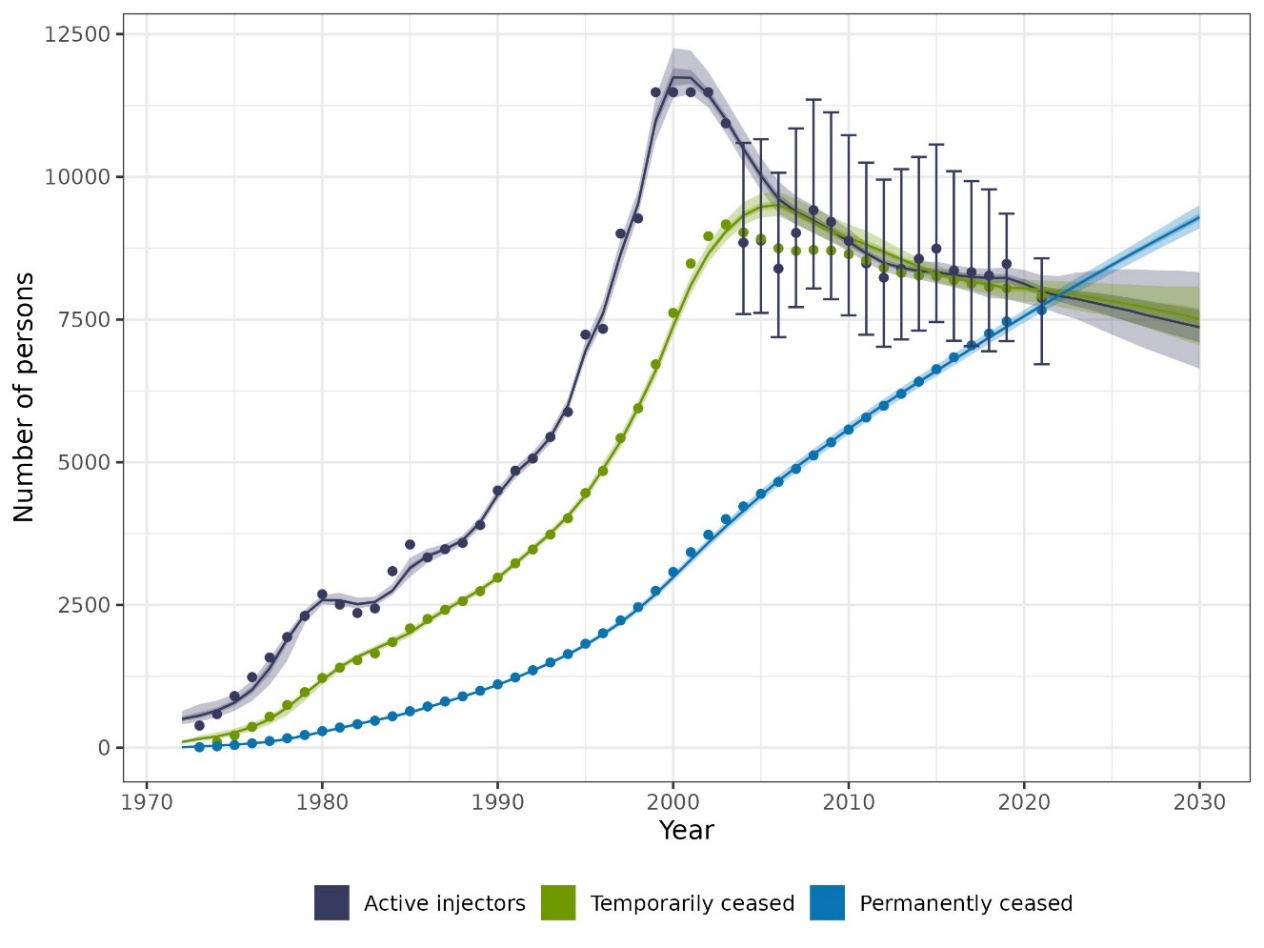


*Figure 3. Estimates of number of people in each of the three subpopulations of people who have injected drugs, and model fit to these estimates, 1972 – 2030.*

# Estimated reduction in the effective infection rate due to NSP and OST

## NSP coverage

The data used to inform the model are based on a survey among active injectors (called ‘BrukerPlan’). BrukerPlan is a tool for municipalities that want to map the extent and nature of drug and mental health problems in people who receive services from the municipality's health and care services. Participants are residents aged 16 and over who are assessed by a professional in the institution as having a drug addiction or psychological problem. Data are registered by health care personnel. From 2016 – 2019, just under 4,000 active injectors answered the survey, and of these the proportion living in a municipality that distributed sterile needles and syringes increased by 15 percentage points (per year 77%, 80%, 87% and 92%) [8]. The notable increase in 2018 and 2019 was likely (at least partially) a consequence of the 2018 clarification by the Norwegian Directorate of Health that municipalities are legally required to freely provide clean needles and syringes to residents in need of such services, under the Infectious Disease Control Act. Using other methods give similar estimates of coverage. For example, based on regular surveys conducted among municipalities in Norway from 2016 – 2021, approximately 87% of the Norwegian population lived in municipalities that freely provided clean needles and syringes. Municipalities that did not were mostly restricted to those with a small population (<20,000 inhabitants). In 2020, among the municipalities that responded to the survey there were 299 overdose deaths (92% of the 342 overdose deaths in Norway that year), of which 96% occurred in municipalities that freely provide clean needles and syringes [8].


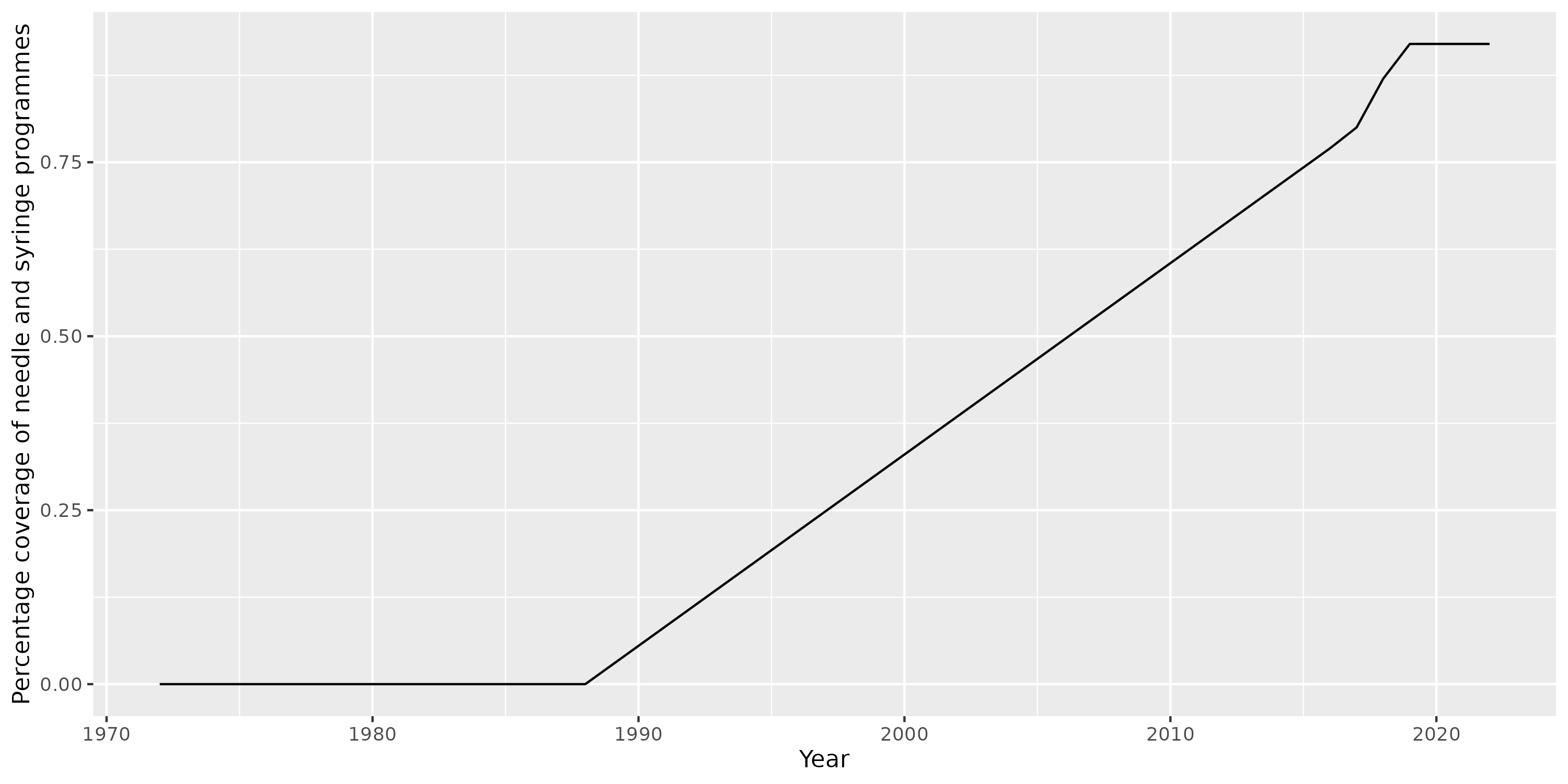


*Figure 4. Estimated coverage of needle and syringe programmes in Norway, 1972 – 2022.*

## Number of patients on OST


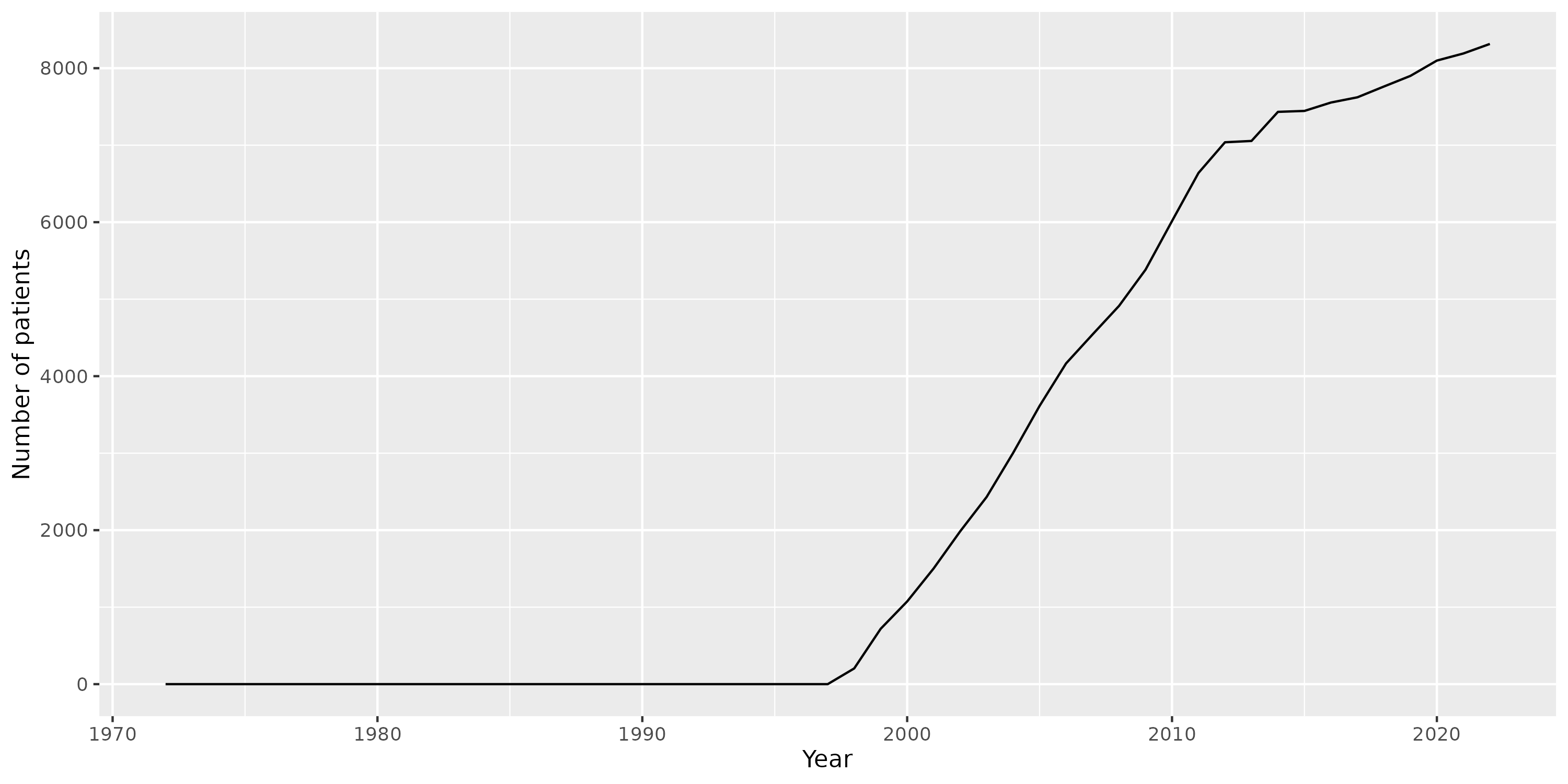


*Figure 5. Number of patients in opioid substitution therapy in Norway, 1972 – 2022.*

## Estimated reduction in the effective infection rate


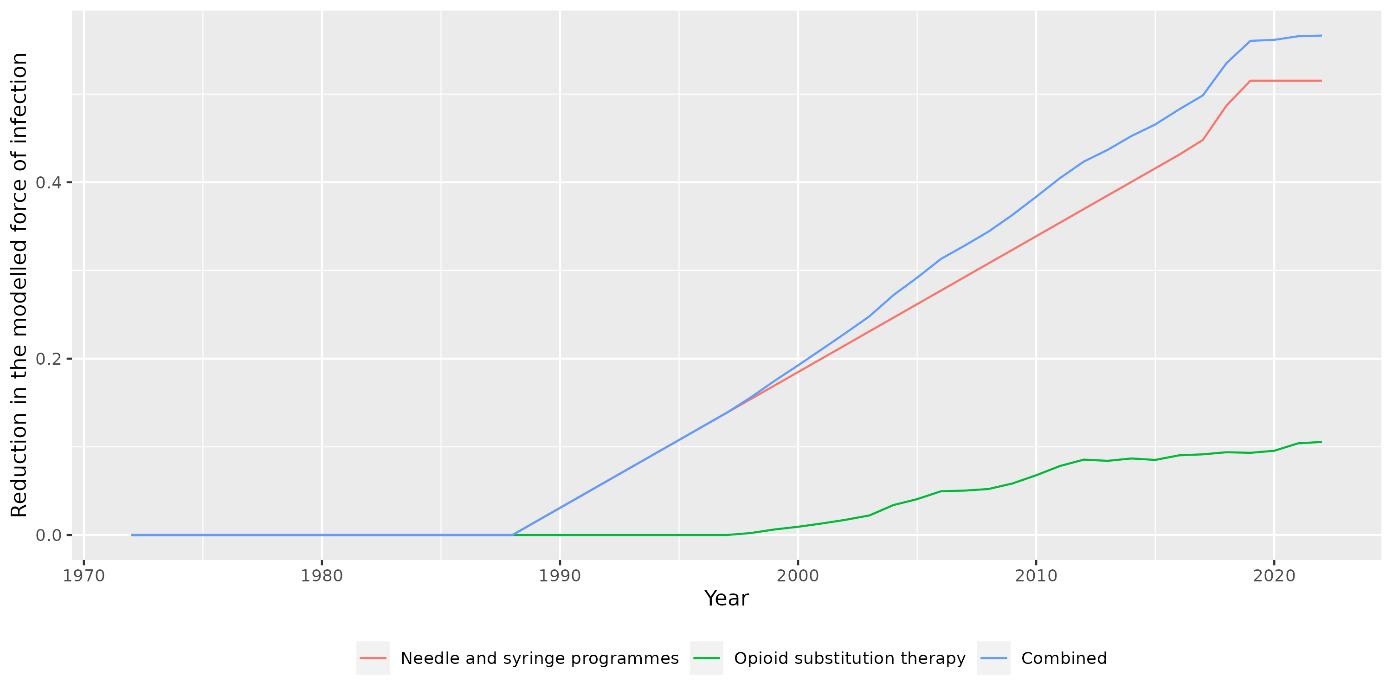


*Figure 6. Estimated reduction in the baseline model infection rate among active PWID from the gradually increasing coverage of needle and syringe programmes and opioid substitution therapy, 1972 – 2022.*

# Gini coefficient

In line with previous modelling work in Norway [3] we used a Gini coefficient, based on the dispersion of overdose deaths in Norway, which was used as a proxy for the geographical dispersion of the injecting epidemic. The Gini coefficient modulated the rate of infection over time. The more disperse the overdose deaths, the lower the transmission rate. The Gini coefficient was calculated for the years 1996 – 2022 from county-level data on yearly number of overdose deaths [9]. We fit an exponentially decaying function to extrapolate this trend to the entire modelled period.


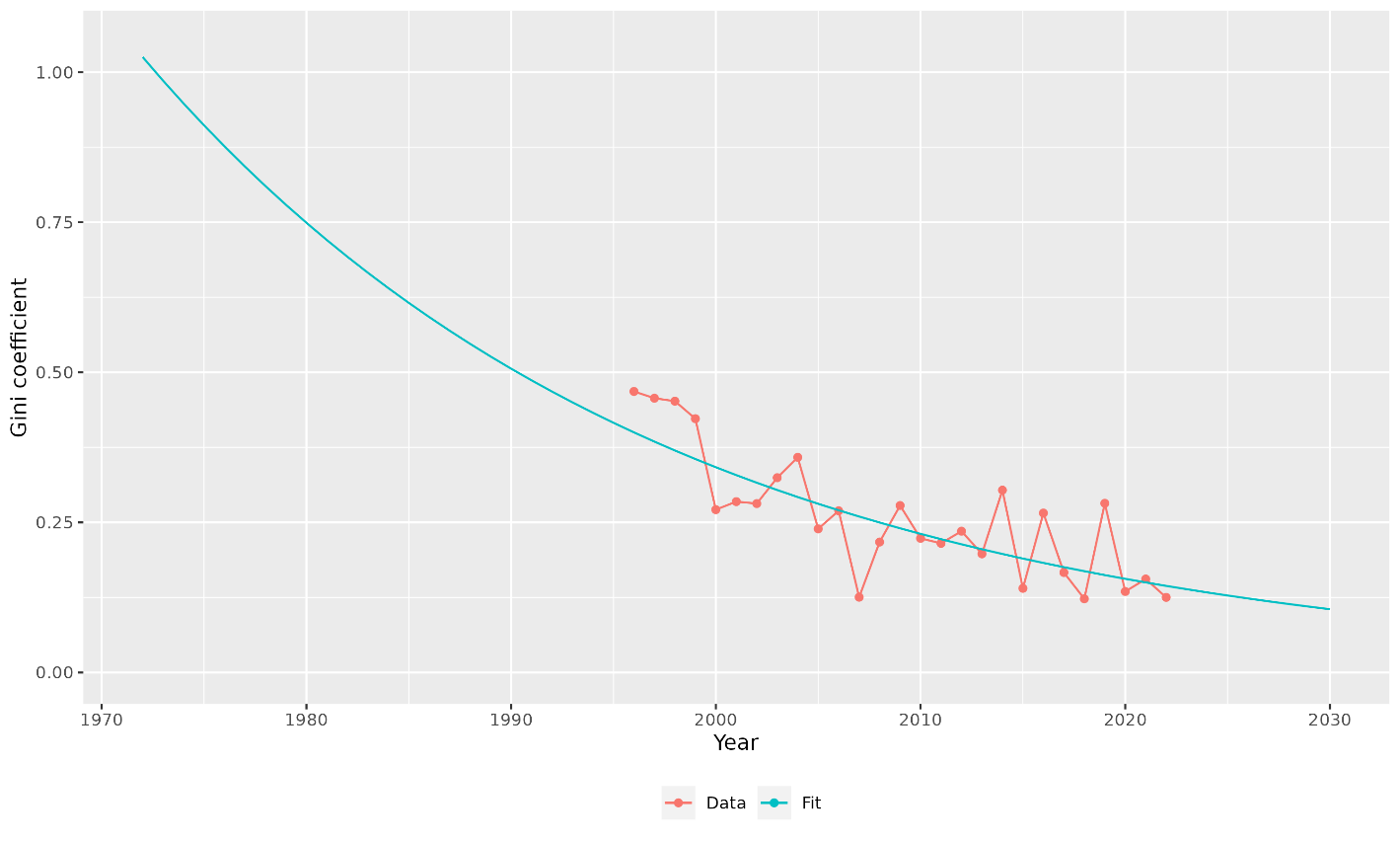


*Figure 7. Gini coefficient for the dispersion of overdose deaths in Norway and fit to data.*

# Model fit to data on HCV antibody prevalence among PWID in Oslo.


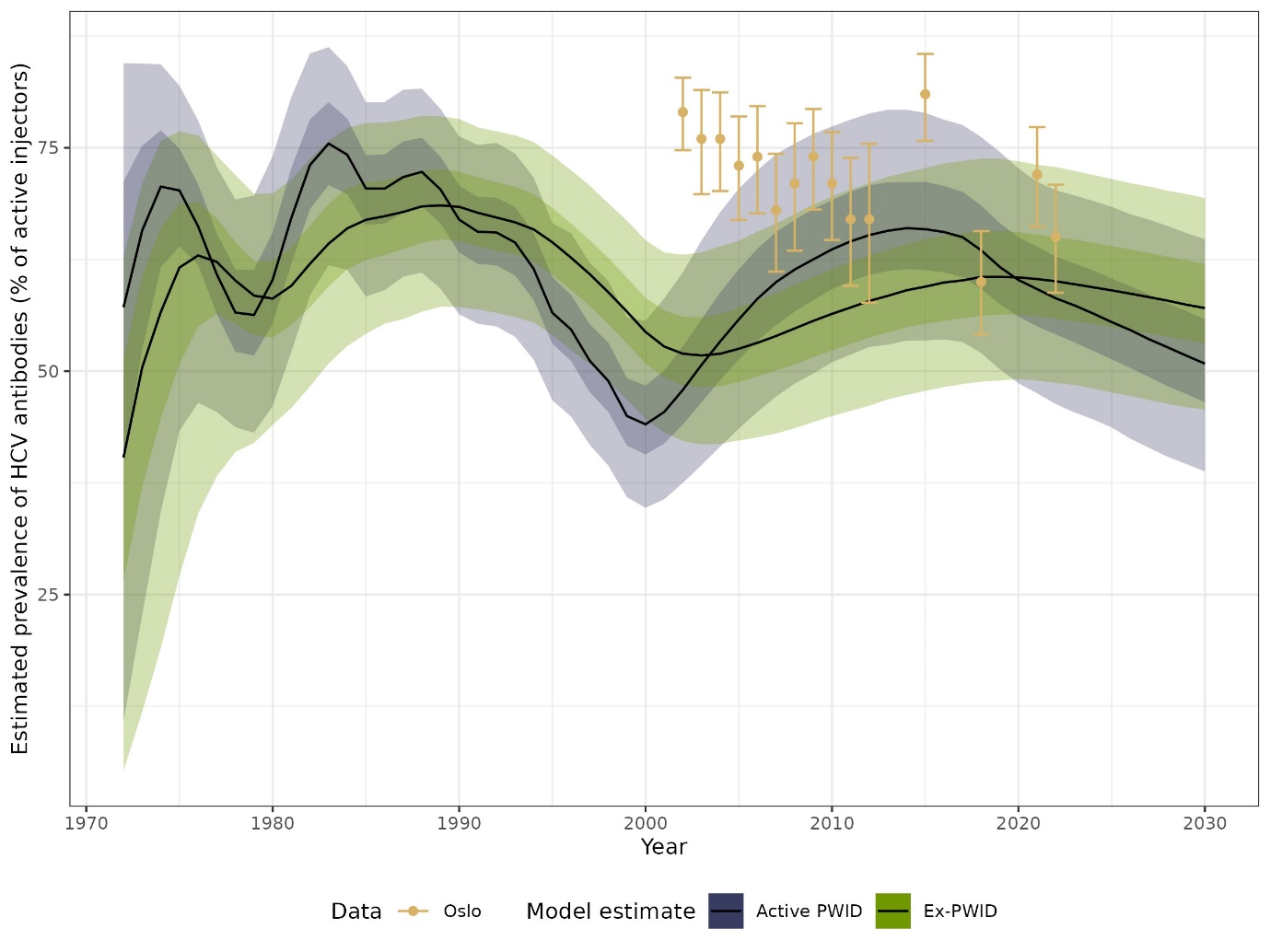


*Figure 8. Model fit to hepatitis C antibody prevalence data from the survey among people who inject drugs attending low threshold health and social care services in Oslo, 1972 – 2022.*

The solid line is the median value, the darker shaded band is the interquartile credible interval, and the lighter shaded band is the 95% credible interval. The trend in model estimates prior to the first data point being available should be interpreted with caution. Furthermore, the antibody data are from Oslo only, so it is more difficult to say how well they can be generalised.

# Estimated net immigration of people with chronic hepatitis C to Norway

We used data from Statistics Norway on the annual number of current residents by country of birth to inform the net immigration from different countries to Norway each year [10]. Annual data are not available from Statistics Norway for the years 1971 – 1979 and 1981 – 1985. No data exist on the prevalence of chronic hepatitis C among different immigrants’ groups in Norway. We assumed that the prevalence among immigrants was the same as in the country of birth, using estimates from the Polaris Observatory [11]. For countries without a national estimate, we used the Global Burden of Disease regional estimates [11]. These data collectively informed the estimated net immigration of people with chronic hepatitis C to Norway, by year and country of birth (Figure 9).


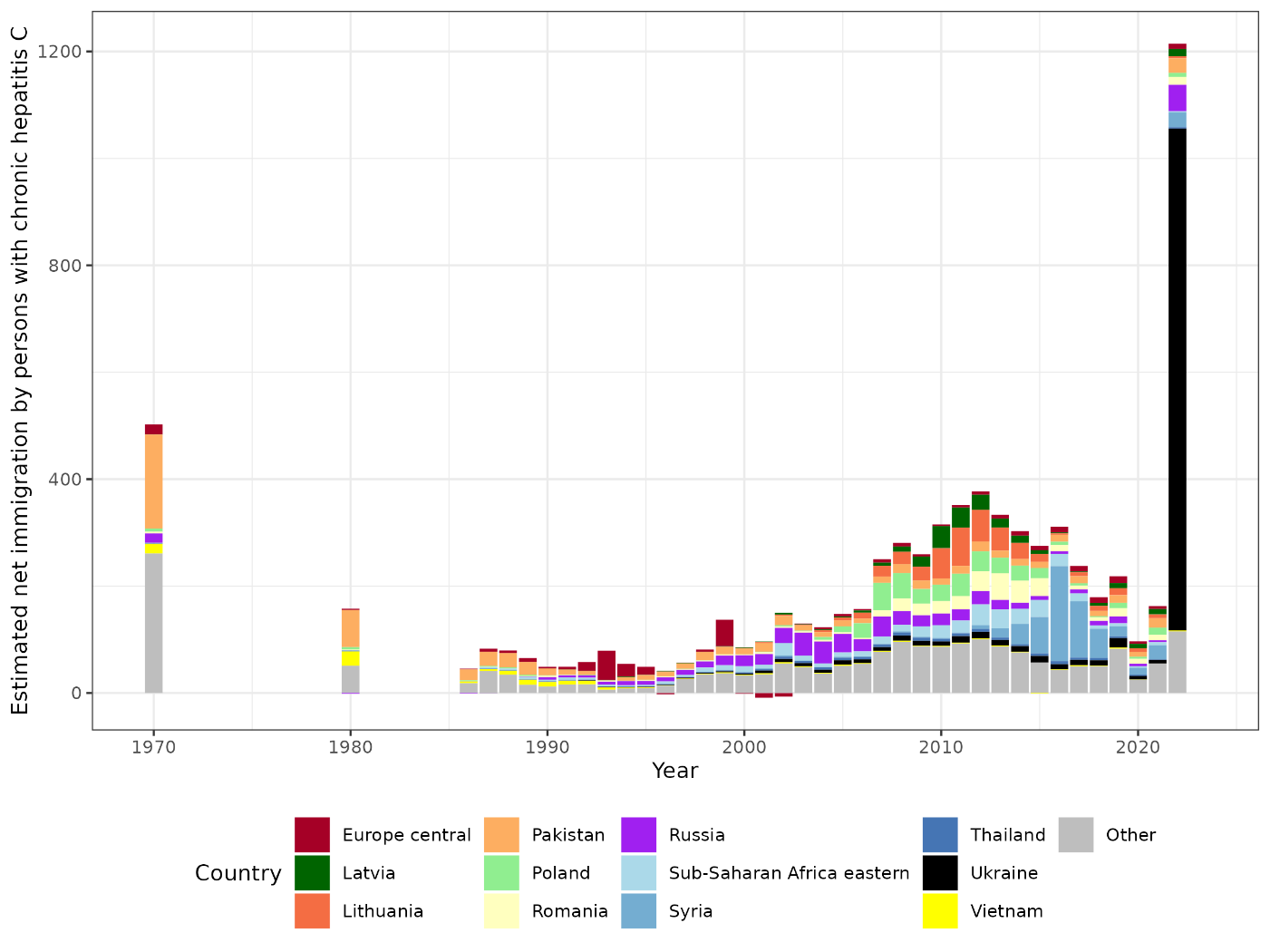


*Figure 9. Estimated net immigration of people with chronic hepatitis C to Norway, by year and country, 1970 – 2022.*

‘Europe central’ includes Albania, Bosnia-Hercegovina, Kosovo, Montenegro and Serbia. ‘Sub-Saharan Africa eastern’ includes Djibouti, Eritrea and Somalia.

# Proportion of PWID in prevalence surveys born outside Norway

Table 1. Number and proportion born outside Norway from prevalence surveys among people who inject drugs attending low threshold health and social care services with reported country of birth, by survey year and location, 2007 – 2022.

| **Survey year** | **Survey location** | **Number of participants born outside Norway (%)** | **Number of HCV RNA positive participants born outside Norway (%)** |
| --- | --- | --- | --- |
| 2007 | Oslo | 26/192 (14%) | 12/96 (13%) |
| 2008 | Oslo | 24/163 (15%) | 10/68 (15%) |
| 2009 | Oslo | 31/246 (13%) | 15/103 (15%) |
| 2010 | Oslo | 35/230 (15%) | 13/92 (14%) |
| 2011 | Oslo | 23/176 (13%) | 10/73 (14%) |
| 2015 | Oslo | 22/267 (8%) | 8/113 (7%) |
| 2015 | Bergen | 10/116 (8%) | ^a^ |
| 2015 – 2017 | Trondheim | ^a^ | 6/108 (6%) |
| 2018 | Oslo | 51/278 (18%) | 9/71 (13%) |
| 2021 | Oslo | 37/257 (14%) | 6/37 (16%) |
| 2022 | Oslo | 42/247 (17%) | 3/22 (14%) |

^a^ The authors did not have access to these data points.

# Treatment input data

The collective estimated number of treatments and treatment success rate per year among the modelled risk groups, active PWID, ex-PWID and immigrants, are presented in Figure 10. The data sources used and assumptions made to calculate these input data are described in the following text.


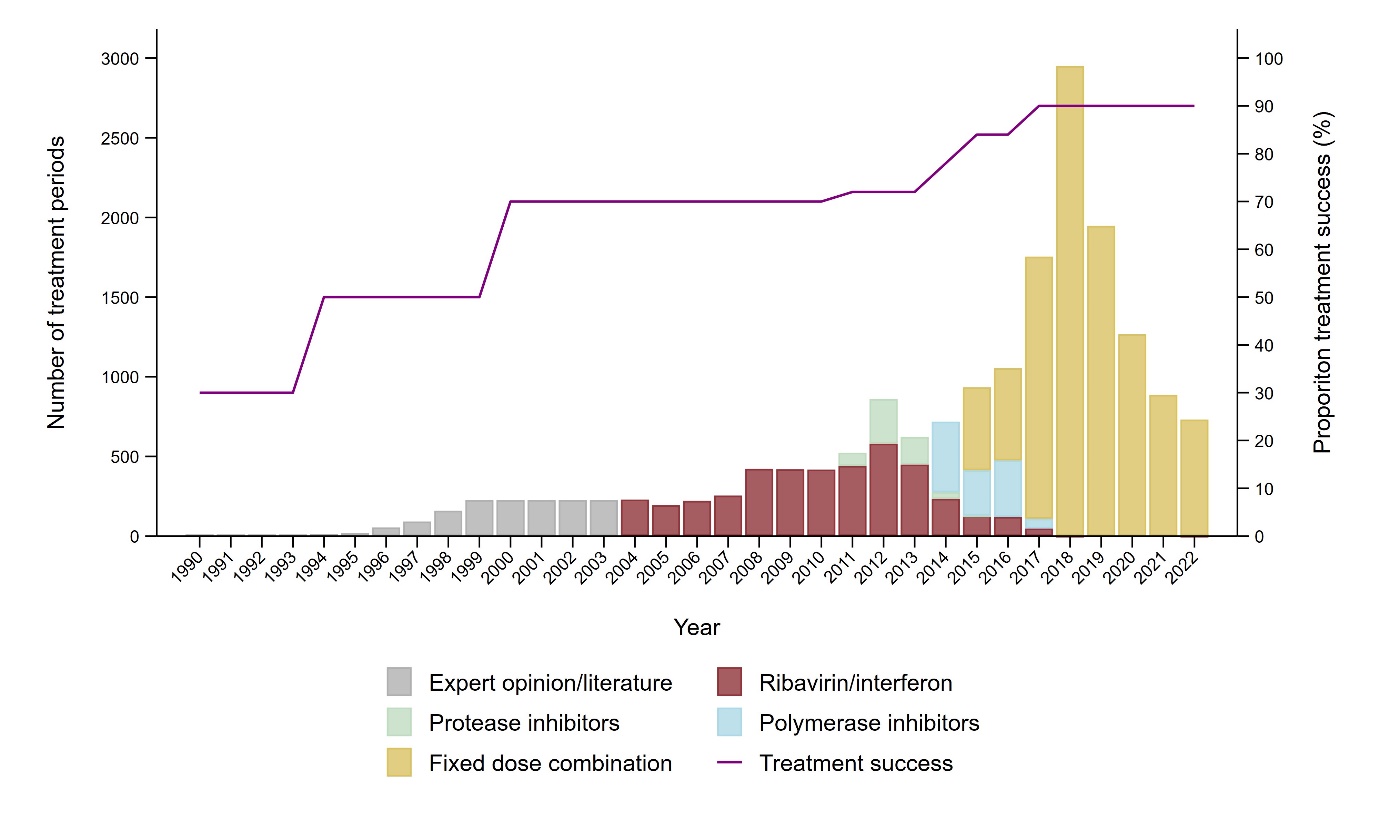


*Figure 10. Estimated number of treatments by class of medication and data source, and estimated treatment success rate among people who inject drugs and immigrants, Norway, 1990 – 2022.*

Other than expert opinion/literature, data come from the Norwegian Prescription Drug Registry. Ribavirin/interferon: Anatomical Therapeutic Chemical (ATC) codes J05AP01, L03AB04, 05, 10 or 11. Treatment periods with ribavirin only are excluded. Protease inhibitors: ATC codes J05AP02 – J05AP05. Polymerase inhibitors: ATC codes J05AP07 – J05AP09. Fixed dose combinations: ATC codes J05AP51 – J05AP57. The definition of treatment periods and description of how treatment success was determined are presented in the text below. For treatment periods where >1 class of medicine was dispensed, these are presented as a) a protease inhibitor, if a protease inhibitor was dispensed and a polymerase inhibitor or fixed dose combination were not dispensed, b) a polymerase inhibitor if a polymerase inhibitor was dispensed and a fixed dose combination was not dispensed, or c) a fixed dose combination if a fixed dose combination was dispensed.

## Number of treatments

For the years 1990 – 1999, we assumed a gradually increasing number of treatments for hepatitis C based on studies in Norway [12-14] as well as expert opinion that approximately half of treatments for hepatitis C in Norway in the 1990s were carried out as part of these studies.

The Norwegian Prescribed Drug Registry (LMR) contains personally identifiable data on medicines dispensed by prescription from pharmacies in Norway since 2004 [15]. As the number of treatment periods (defined below) in 2004 identified in LMR matched the number estimated for 1999 based on expert opinion and studies in Norway, we assumed that the number of treatments remained stable for the years 2000 – 2003.

From 2004 – 2022, we used data from LMR to calculate the number of treatment courses and individuals treated for hepatitis C, defined by the Anatomical Therapeutic Chemical (ATC) codes J05AP (antivirals for treatment of HCV infections) and L03AB04, 05, 10 or 11 ((peg-)interferon-α) [16]. The length of treatment for hepatitis C has ranged from 8 – 72 weeks [17-22]. Persons infected with hepatitis C may also undergo several treatment periods, either due to treatment failure or reinfection. Therefore, we aggregated prescriptions into treatment periods per person, based on the month of the prescription and medicine dispensed.

A treatment period included prescriptions where:

- There was ≤1 month between prescriptions, except when a new class of direct acting antiviral (DAA) was dispensed for the first time (protease inhibitors (ATC code J05AP02 – J05AP05), polymerase inhibitors (J05AP07 – J05AP09) or fixed dose combinations (J05AP51 – J05AP57)).
- There were 2 – 3 months between prescriptions, but no change in the medicines dispensed.
- There were ≤6 months between prescriptions, when interferon and/or ribavirin (J05AP01) were the only medicines dispensed and prior to 2014. This is because the dosing and frequency of prescriptions for these medicines could vary, until 2014 from which time interferon and ribavirin were only recommended to be prescribed in 12-week courses to specific patients [18].
- Continued prescription of ribavirin and/or interferon, following a treatment period with a DAA, were included as part of the previous DAA treatment period.

After aggregating treatment periods using the algorithm above, any periods where ribavirin was the only medicine dispensed were dropped.

The model does not include persons who do not belong to the risk groups PWID or immigrants. Previous studies have found that a small proportion of hepatitis C cases in Norway are among persons in risk groups other than PWID or immigrants [23-25]. However, treatment of these persons also needs to be considered. To do this we used data from LMR liked to notified cases of hepatitis C in the Norwegian Surveillance System for Communicable Diseases (MSIS). Among 13,100 notified cases (including 41 (0.3%) registered reinfections, i.e. individuals registered twice) who had received treatment from 2004 – 2022, 3,711 (38%) were Norwegian-born but with unknown route of transmission, and a further 291 (2.2%) had unknown country of birth. Of the 9,098 (69%) remaining individuals who were either born in Norway with reported route of transmission or born overseas, 562 (6.2%) were reported to have been born in Norway and infected by another route of transmission than injecting drug use. By year of first treatment, this ranged from an average 9.0% (n=188/2,101) in the years before the availability of interferon-free DAA (2004 – 2013) to 6.7% (153/2,259) in the interferon-free DAA years before treatment was first made available for all infected (2014 – 2017) to 4.6% (n=221/4,738) in the treatment for all era (2018 – 2022). We therefore reduced the number of treatments by 9% per year from 1990 – 2013, 7% per year from 2014 – 2017 and 5% per year from 2018 – 2022, to account for treatments given to persons in risk groups other than those modelled. The higher proportional reduction in earlier years is logical, given that treatment uptake among active injecting drug users in Norway was low before interferon-free DAA were made available for all [26].

We based the rate of treatment uptake for active PWID from 2010 – 2019 on a registry-based study among PWID attending low threshold health and social care services in Oslo [26]. Based on this study and the trend in the overall number of treatments, from 1990 – 2003 we assumed a gradually increasing rate of uptake among active PWID to 0.34 per 100 person-years, and thereafter a stable rate until 2009. For 2020 – 2022, we assumed the same rate of uptake as in 2019 (22.1 per 100 person-years) (Figure 11). For the remaining treatments not allocated to active PWID, we assume that the per-person annual probability that ex-PWID or immigrants will seek treatment is the same. This probability was varied each year by the model, so the total number of treatments matched the data, although we constrained the variation so that the model could not apply an unrealistically high treatment rate (see further details under ‘Model fit’ above). The model fit to the number of treatments in the input data is presented in Figure 12.


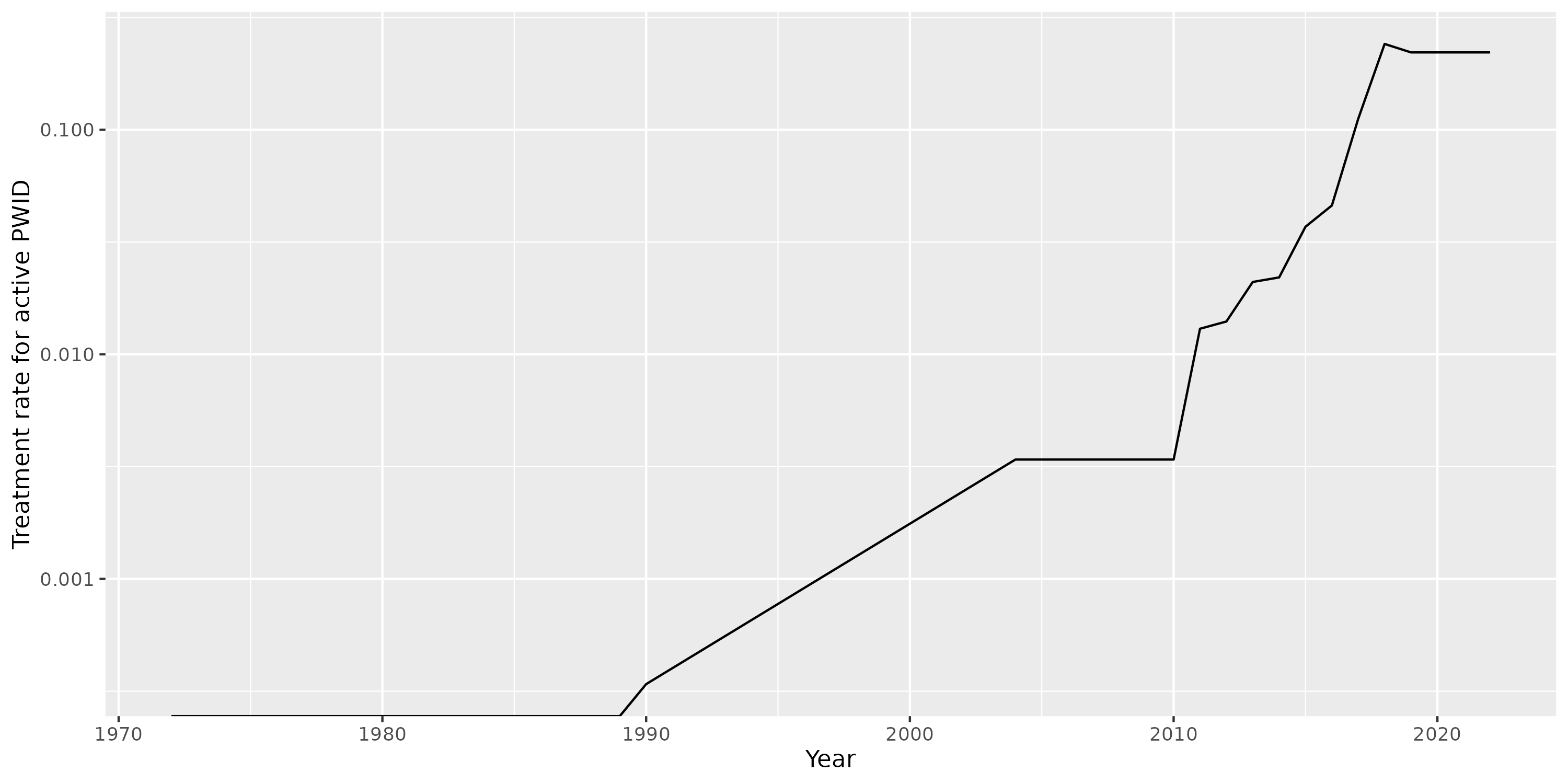


*Figure 11. Estimated rate of treatment uptake for active PWID, based on [26], by year, 1972 – 2022.*


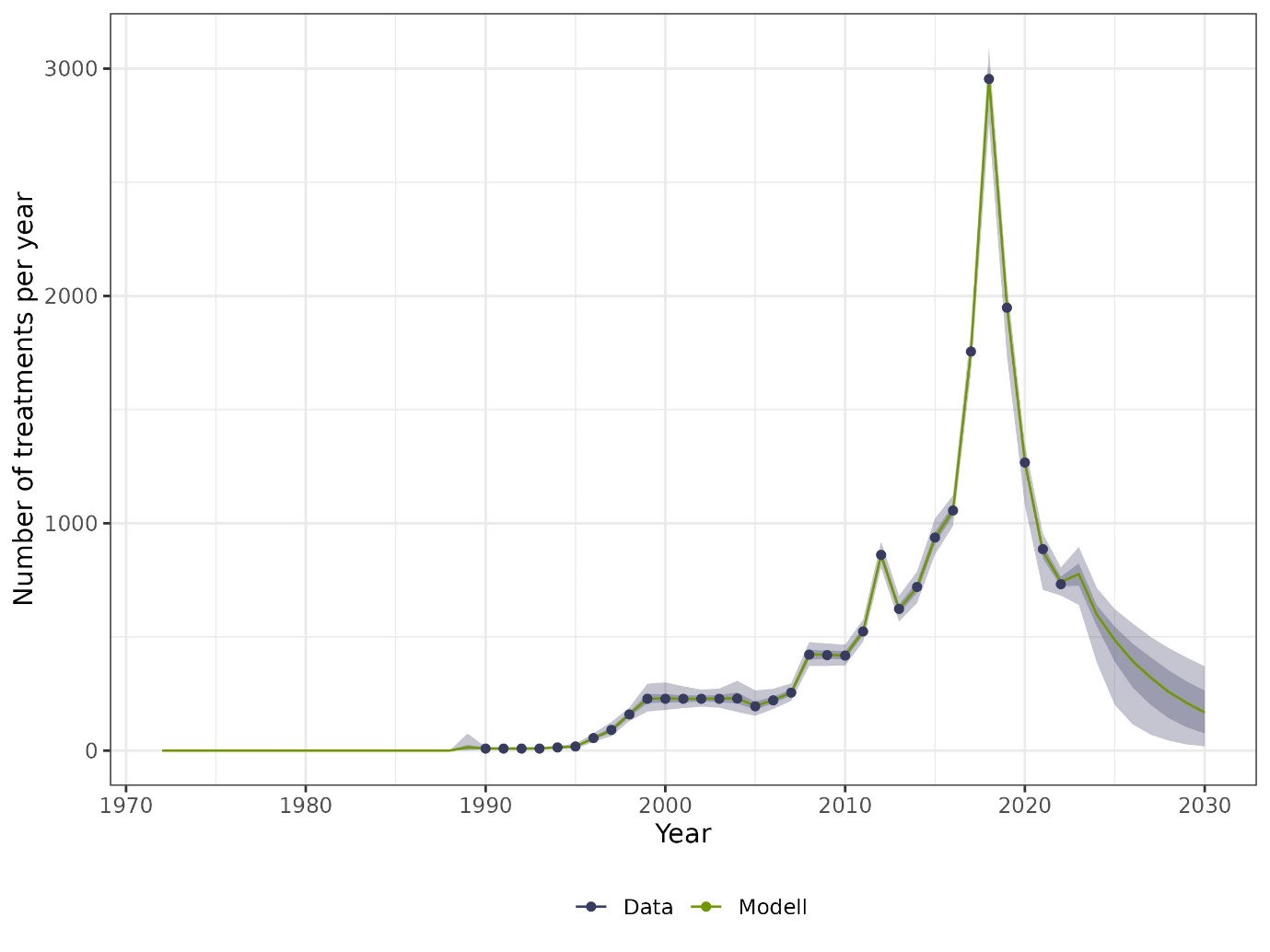


*Figure 12. Model fit to the number of treatments in the input data.*

## Treatment success

For the period 1990 – 2010 (ribavirin/interferon), we assumed a gradually increasing treatment success rate from 30 – 70 % based on studies in Norway [12-14, 27, 28].

For each treatment period (as defined above) with the prescription of at least one DAA, we determined the number and proportion of DAA treatment courses per year (2011 – 2022) where complete treatment was dispensed, based on the DAA(s) and number of Defined Daily Dose (DDD) prescribed in the period, according to Norwegian clinical guidelines [18-22]. The data from this analysis will be published in full elsewhere, but a similar analysis on 2011 – 2018 data is available in [29].

Based on the data on treatment completion and compilation of different treatment types (Figure 10), we took a pragmatic approach to increasing the treatment success rate from 2011 onwards. We increased the treatment success rate to 72% in 2011 – 2013, based on evidence of an approximate 30% increase in treatment success among genotype 1 patients who received protease inhibitors [30-32]. From 2014, when interferon-free DAA became available, we increased treatment success to 78% (2014), 84% (2015 and 2016) and 90% (from 2017). This was to reflect 1) gradually improving treatment effectiveness [30] with the increasing use of fixed dose combination antivirals (Figure 10), 2) varying treatment effect by genotype and degree of liver disease [33], parameters we did not have data on, 3) increasing treatment completion rates [29], 4) evidence of treatment success despite suboptimal adherence for interferon-free direct acting antivirals [34, 35] and 5) gradual relaxing of treatment indications which culminated in all hepatitis C infected persons being eligible for treatment in Norway, free of charge, from 1 February 2018 [36].

The treatment success rate was kept constant across the different risk groups in the model. This is supported by evidence of high treatment success rates among PWID in Oslo [37], high treatment completion and success rates from systematic reviews and meta analyses among PWID [38, 39] and evidence of treatment success despite suboptimal adherence for interferon-free direct acting antivirals [34, 35].

# Counterfactual scenarios and sensitivity analyses

All counterfactual scenarios and sensitivity analyses are described in Table 2 and results are presented in Figure 13, Figure 14 and Table 6 – Table 10. Results in all these analyses were generally consistent with the baseline model with largely overlapping estimates or varied as expected. Some examples include assuming larger/smaller PWID populations resulting in larger/smaller absolute and relative changes in incidence and prevalence estimates. There was a slightly higher incidence among active PWID when assuming a lower effect of NSP and a notably higher incidence when excluding the Gini coefficient. Excluding the Gini coefficient also gave slightly higher prevalence estimates among active PWID towards the end of the study period. Assuming no leakage of immigrants with chronic hepatitis C to active PWID resulted in higher prevalence among immigrants, and consequently ex-PWID owing to the assumption that the per-person annual probability of seeking treatment in these two groups was the same. Assuming a lower rate of treatment uptake among active PWID resulted in marginally higher absolute prevalence among active PWID, but lower absolute prevalence among ex-PWID and immigrants. Assuming a collectively more pessimistic scenario (higher input data on prevalence among active PWID, lower reduction in the risk of infection for NSP and OST, lower overall number of treatments and lower treatment uptake among active PWID) increased the estimated incidence among active PWID and prevalence among all risk groups. In all sensitivity analyses, the incidence in 2022 was consistently below the WHO absolute target (2 per 100). While results from these sensitivity analyses did not challenge the conclusions, they provide useful information on how much estimates vary under a different set of assumptions.

*Table 2: Summary of counterfactual scenarios and sensitivity analyses*

| **Number** | **Baseline model** | **Counterfactual scenario/sensitivity analysis** |
| --- | --- | --- |
| 1 | Size of PWID population based on point estimates in data from The Norwegian Institute of Public Health. | Assumed a 15% smaller population per year (a).  Assumed a 15% larger population per year (b). |
| 2 | Surveys among PWID attending low threshold health and social care services in Oslo, Bergen, Stavanger and Trondheim from 2002 – 2022 are representative of the prevalence among active PWID nationally. | Not all participants in these surveys reported recent injecting, thus we varied the cohorts’ size and prevalence, based on available data on recent injecting drug use^a^. |
| 3 | The average time spent in compartment for acute infection is 6 months and 26% [40] of transitions out of the compartment for acute infection go to clearing infection while the rest become chronic. | Clearance rate of acute infection 20% (a).  Clearance rate of acute infection 30% (b).  The average time spent in compartment for acute infection is 12 months and 36% [40] of transitions out of the compartment for acute infection go to clearing infection while the rest become chronic (c). |
| 4 | NSP reduces the risk of hepatitis C virus infection by 56% (point estimate from literature [41]). | NSP reduces the risk of infection by 20% (lower 95% confidence interval from literature [41]). |
| 5 | OST reduces the risk of hepatitis C virus infection by 50% (point estimate from literature [41]). 20% of patients on OST per year [42] overlap with active PWID. | OST reduces the risk of infection by 37% (lower 95% confidence interval from literature [41]) (a).  30% of patients on OST per year overlap with active PWID (b). |
| 6 | Prevalence of chronic hepatitis C among immigrants based on point estimates from the Polaris Observatory [11]. | Prevalence estimates based on middle value between lower 95% confidence interval and point estimate in [11] (a).  1% prevalence among immigrants from Ukraine (b). |
| 7 | Gradual leakage of immigrants with chronic hepatitis C to active PWID such that immigrants constituted 10% of active PWID with chronic hepatitis C in 2022^b^. | No gradual leakage. |
| 8 | Based on linked registry data, we reduced the number of treatments nationally by 9% per year from 1990 – 2013, 7% per year from 2014 – 2017 and 5% per year from 2018 – 2022 to account for treatments given to persons in risk groups other than those modelled^c^. | Reduced the number of treatments nationally by 10% per year. |
| 9 | Treatment uptake among active PWID with chronic hepatitis C based on [26]. For the remaining treatments, the per-person probability that ex-PWID or immigrants seek treatment is the same. | Same per-person probability of treatment uptake in all groups^d^ (a).  In [26] 73% of participants were currently on OST, which was associated with increased treatment rates. Also, the authors reported potential overestimation of treatment rates for 2017 – 2019. We therefore reduced treatment rate among active PWID from [26] by 25% in 2017 – 2022 (b). |

*Table 2. continued*

| **Number** | **Baseline model** | **Counterfactual scenario/sensitivity analysis** |
| --- | --- | --- |
| 10 | Rate of treatment uptake and success based on published studies, data from the Norwegian Prescribed Drug Registry and data on prescriptions from hospital pharmacies^c^. | Assumed same rate of treatment uptake from 2013 onwards^e^ (a).  Assumed same rate of treatment uptake and success from 2013 onwards^e^ (b). |
| 11 | Coverage of NSP based on data from the Centre for Alcohol and Drug Research [8]. Rate of treatment uptake among active PWID with chronic hepatitis C based on [26]. | To examine to effect of the COVID-19 pandemic, NSP coverage decreased to 70% and reduced treatment rate among active PWID from [26] by 25%, in 2020 and 2021. |
| 12 | Gini coefficient reduces the infection rate over time to reflect the increasing geographical spread in the injecting epidemic. | No Gini coefficient. |
| 13 | - | A combination of nr. 2, 4, 5a, 8 and 9b, to investigate a more pessimistic scenario with a lower reduction in the risk of infection for NSP and OST, lower overall number of treatments and lower treatment uptake among active PWID. |
| 14 | Other model parameters. | Mortality rate standard deviation of 10 for the age distribution estimation, instead of the standard deviation of 5 in the baseline model (a).  Overdose mortality rate among active PWID 0.04 per PWID, instead of 0.03 in the baseline model (b).  Removing posterior draws where the total log-likelihood is below -2500 (c). |

NSP: Needle and syringe programmes. OST: Opioid substitution therapy. PWID: People who inject drugs.

^a^ In the baseline model, we assume that the surveys among PWID are representative of the prevalence among active PWID nationally, see <https://github.com/folkehelseinstituttet/hepatitis_C_model/> for these input data. Not all participants in these surveys reported recent injecting, thus we varied the size of the cohort and HCV RNA prevalence (Table 3), based on data available to the authors on recent injecting (Table 4 and Table 5), to explore the sensitivity of results to this assumption. We based the relative change in cohort size from 2002 – 2009 on 2009 data, as the method of recruitment in Oslo was consistent in this period. We increased the relative change in cohort size in 2012 and 2015 from 5% to 10 %, as recruitment in Oslo changed to include payment for participation, which may have recruited more non-recent injectors. We applied the same correction factor to the size of the 2015 cohort in Bergen, where the method of recruitment was similar to Oslo. For the Bergen/Stavanger cohort, the study coordinators informed that 60% had reported injecting drug use in the last 6 months. For Trondheim, the relative change in cohort size is based on reported data in [43]. For the absolute percentage change in HCV RNA prevalence, we increased this in all surveys by 5% (except 2022 in Oslo), based on data from Oslo.

^b^ Informed by the data in Table 1.

^c^ As described in the section ‘Treatment input data’ above.

^d^ This in effect means a higher treatment rate among active PWID and a lower treatment rate among ex-PWID and immigrants, compared to baseline.

^e^ Model also not fitted to HCV RNA prevalence data from surveys among PWID attending low threshold health and social care services from 2013 onwards.

*Table 3**: Correction factor applied to the size of the cohort and HCV RNA prevalence among surveys of people who inject drugs attending low threshold health and social care services in Oslo, Bergen, Stavanger and Trondheim for model number 2^a^.*

| **Survey year** | **Survey location** | **Relative change in cohort size** | **Absolute % change in HCV RNA prevalence** |
| --- | --- | --- | --- |
| 2002 | Oslo | * 0.95 | +5 |
| 2003 | Oslo | * 0.95 | +5 |
| 2004 | Oslo | * 0.95 | +5 |
| 2005 | Oslo | * 0.95 | +5 |
| 2006 | Oslo | * 0.95 | +5 |
| 2007 | Oslo | * 0.95 | +5 |
| 2008 | Oslo | * 0.95 | +5 |
| 2009 | Oslo | * 0.95 | +5 |
| 2010 | Oslo | * 0.95 | +5 |
| 2011 | Oslo | * 0.95 | +5 |
| 2012 | Oslo | * 0.90 | +5 |
| 2015 | Oslo | * 0.90 | +5 |
| 2015 | Bergen | * 0.90 | +5 |
| 2016 | Trondheim | * 0.94 | +5 |
| 2017 | Bergen/Stavanger | * 0.60 | +5 |
| 2018 | Oslo | * 0.80 | +5 |
| 2020 | Bergen/Stavanger | * 0.60 | +5 |
| 2021 | Oslo | * 0.80 | +5 |
| 2022 | Oslo | * 0.85 | No change |

^a^ See Table 2 for further details.

Table 4: Number and proportion of people who inject drugs attending low threshold health and social care services in Oslo with self-reported recent injecting drug use, by survey year.

| **Survey year** | **Injected in last 4 weeks (%)** | **Injected in last 12 months (%)** |
| --- | --- | --- |
| 2009^1^ | 220/250 (88%) | *Not asked* |
| 2018 | 199/267 (75%) | *Not asked* |
| 2021 | 202/257 (79%) | *Not asked* |
| 2022 | 207/247 (84%) | 222/247 (90%) |

^1^ Heroin only. The proportion who had injected any substance in the last 4 weeks was likely higher. The authors did not have access to these data, nor data for other survey years.

Table 5: Number and proportion of people who inject drugs attending low threshold health and social care services in Oslo who were HCV RNA positive, by survey year and self-reported recent injecting drug use.

| **Survey year** | **Injected in last 4 weeks (%)** | **Not injected in last 4 weeks (%)** |
| --- | --- | --- |
| 2018 | 63/199 (32%) | 8/68 (12%) |
| 2021 | 36/202 (18%) | 1/55 (2%) |
| 2022 | 18/207 (9%) | 4/40 (10%) |


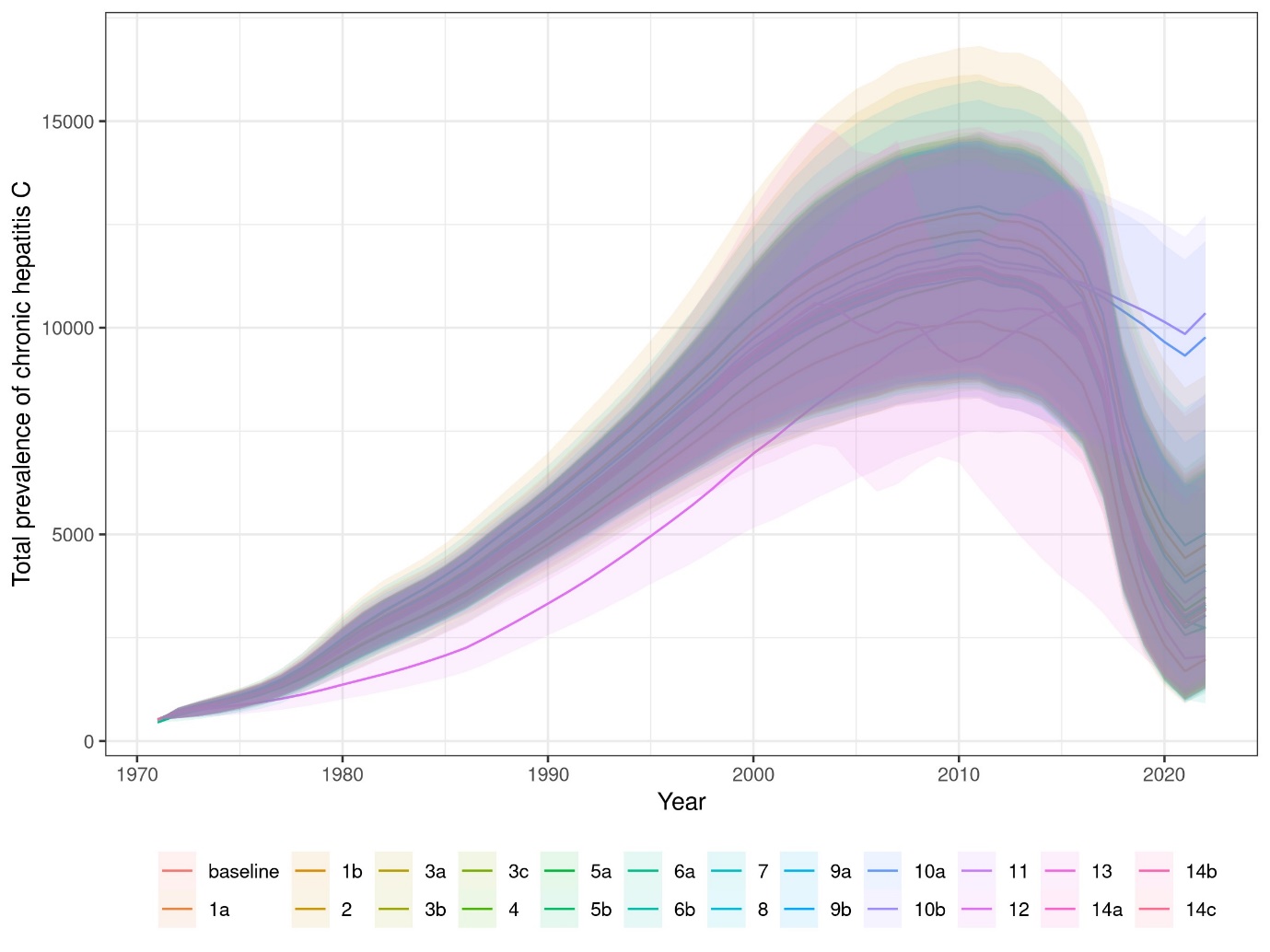


*Figure 13.* ***Prevalence*** *of chronic hepatitis C in the baseline model, counterfactual scenarios and sensitivity analyses, by year and model number^a^,* ***overall****, Norway, 1972 – 2022.*

^a^ See Table 2 for further details.


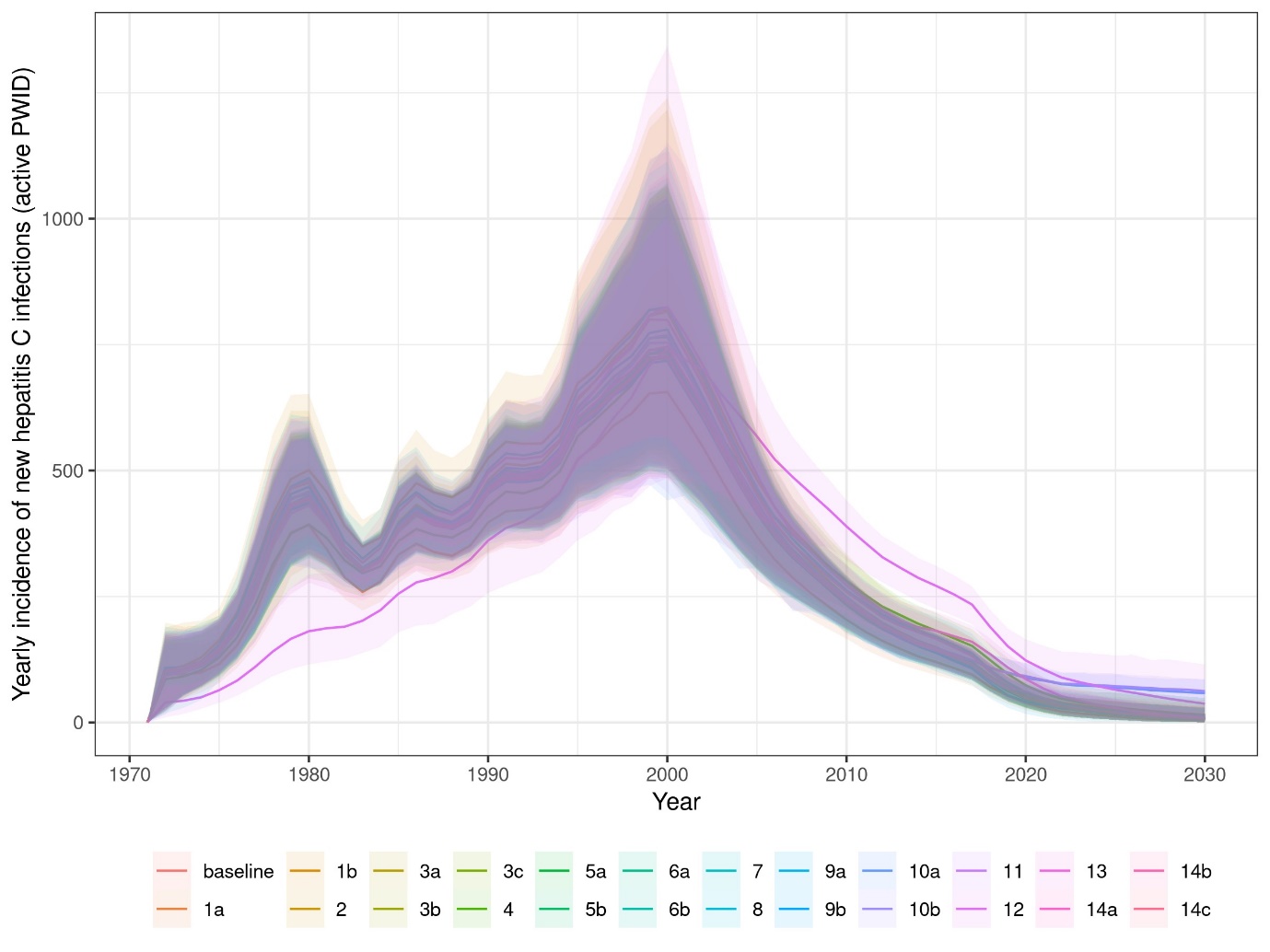


*Figure 14.* ***Incidence*** *of hepatitis C in the baseline model, counterfactual scenarios and sensitivity analyses, by year and model number^a^,* ***active PWID****, Norway, 1972 – 2030.*

^a^ See Table 2 for further details.

*Table 6: Results for the* ***prevalence*** *of chronic hepatitis C in the baseline model, counterfactual scenarios and sensitivity analyses,* ***overall****.*

| Model number^a^ | Peak prevalence year | Peak prevalence (95% CrI) | Prevalence in 2015 (95% CrI) | Prevalence in 2022 (95% CrI) | Relative change in prevalence, 2022 compared to 2015 |
| --- | --- | --- | --- | --- | --- |
| Baseline | 2011 | 11,306 (8,743 – 14,617) | 10,395 (7,786 – 13,743) | 3,202 (1,273 – 6,601) | 0.31 (0.16 – 0.48) |
| 1a | 2011 | 10,153 (8,278 – 12,557) | 9,226 (7,318 – 11,572) | 1,976 (1,206 – 4,493) | 0.21 (0.16 – 0.39) |
| 1b | 2011 | 12,777 (9,102 – 16,823) | 11,888 (8,135 – 15,977) | 4,742 (1,362 – 8,856) | 0.40 (0.17 – 0.55) |
| 2 | 2011 | 12,347 (9,113 – 16,135) | 11,431 (8,199 – 15,188) | 4,279 (1,374 – 8,181) | 0.37 (0.17 – 0.54) |
| 3a | 2011 | 11,282 (8,674 – 14,654) | 10,385 (7,720 – 13,749) | 3,170 (1,279 – 6,722) | 0.31 (0.17 – 0.49) |
| 3b | 2011 | 11,427 (8,760 – 14,750) | 10,507 (7,782 – 13,829) | 3,299 (1,295 – 6,762) | 0.31 (0.17 – 0.49) |
| 3c | 2011 | 11,354 (8,679 – 14,595) | 10,438 (7,746 – 13,678) | 3,229 (1,270 – 6,604) | 0.31 (0.16 – 0.48) |
| 4 | 2011 | 11,192 (8,491 – 14,386) | 10,437 (7,694 – 13,674) | 3,485 (1,348 – 6,961) | 0.33 (0.18 – 0.51) |
| 5a | 2011 | 11,323 (8,614 – 14,533) | 10,397 (7,657 – 13,666) | 3,194 (1,274 – 6,545) | 0.31 (0.17 – 0.48) |
| 5b | 2011 | 11,440 (8,817 – 14,704) | 10,500 (7,832 – 13,786) | 3,301 (1,284 – 6,630) | 0.31 (0.16 – 0.48) |
| 6a | 2011 | 11,330 (8,718 – 14,398) | 10,259 (7,622 – 13,316) | 2,750 (1,149 – 6,044) | 0.27 (0.15 – 0.45) |
| 6b | 2011 | 11,393 (8,741 – 14,591) | 10,455 (7,760 – 13,672) | 2,735 (913 – 6,099) | 0.26 (0.12 – 0.45) |
| 7 | 2011 | 12,940 (9,673 – 15,988) | 12,118 (8,777 – 15,230) | 5,026 (1,606 – 8,385) | 0.41 (0.18 – 0.55) |
| 8 | 2011 | 11,372 (8,531 – 14,458) | 10,436 (7,606 – 13,605) | 3,224 (1,245 – 6,491) | 0.31 (0.16 – 0.48) |
| 9a | 2011 | 12,131 (9,030 – 15,519) | 11,294 (8,077 – 14,631) | 4,130 (1,231 – 7,555) | 0.37 (0.15 – 0.52) |
| 9b | 2011 | 11,216 (8,830 – 14,519) | 10,281 (7,850 – 13,622) | 3,035 (1,424 – 6,476) | 0.30 (0.18 – 0.48) |
| 10a | 2011 | 11,799 (8,298 – 14,072) | 11,217 (7,555 – 13,525) | 9,770 (6,153 – 12,099) | 0.87 (0.81 – 0.89) |
| 10b | 2011 | 11,634 (8,342 – 13,986) | 11,210 (7,617 – 13,490) | 10,354 (6,680 – 12,722) | 0.92 (0.88 – 0.94) |
| 11 | 2011 | 11,321 (8,786 – 14,531) | 10,390 (7,867 – 13,656) | 3,202 (1,354 – 6,598) | 0.31 (0.17 – 0.48) |
| 12 | 2013 | 10,472 (7,518 – 14,785) | 10,092 (7,091 – 14,384) | 3,726 (1,437 – 8,415) | 0.37 (0.20 – 0.59) |
| 13 | 2016 | 10,615 (3,575 – 13,364) | 10,463 (3,952 – 13,340) | 2,057 (1,582 – 2,595) | 0.20 (0.40 – 0.19) |
| 14a | 2011 | 11,486 (8,698 – 14,865) | 10,537 (7,711 – 13,928) | 3,345 (1,271 – 6,826) | 0.32 (0.16 – 0.49) |
| 14b | 2011 | 11,351 (8,937 – 14,336) | 10,348 (7,932 – 13,331) | 3,058 (1,280 – 6,149) | 0.30 (0.16 – 0.46) |
| 14c | 2011 | 11,295 (8,890 – 14,434) | 10,381 (7,930 – 13,522) | 3,191 (1,312 – 6,404) | 0.31 (0.17 – 0.47) |

CrI: Credible interval. NSP: Needle and syringe programmes. OST: Opioid substitution therapy. PWID: People who inject drugs. ^a^ See Table 2 for further details.

*Table 7: Results for the* ***prevalence*** *of chronic hepatitis C in the baseline model, counterfactual scenarios and sensitivity analyses,* ***active PWID****.*

| Model number^a^ | Peak prevalence year | Peak prevalence (95% CrI) | Prevalence in 2015 (95% CrI) | Prevalence in 2022 (95% CrI) | Relative change in prevalence, 2022 compared to 2015 |
| --- | --- | --- | --- | --- | --- |
| Baseline | 2008 | 4,342 (3,297 – 5,709) | 3,784 (2,867 – 4,914) | 940 (486 – 1,583) | 0.25 (0.17 – 0.32) |
| 1a | 2008 | 3,892 (3,116 – 4,897) | 3,353 (2,696 – 4,185) | 700 (443 – 1,171) | 0.21 (0.16 – 0.28) |
| 1b | 2008 | 4,932 (3,473 – 6,580) | 4,307 (3,006 – 5,698) | 1,218 (536 – 2,034) | 0.28 (0.18 – 0.36) |
| 2 | 2008 | 4,775 (3,455 – 6,349) | 4,132 (2,993 – 5,422) | 1,150 (550 – 1,909) | 0.28 (0.18 – 0.35) |
| 3a | 2008 | 4,329 (3,273 – 5,755) | 3,768 (2,831 – 4,922) | 926 (488 – 1,620) | 0.25 (0.17 – 0.33) |
| 3b | 2008 | 4,388 (3,321 – 5,765) | 3,806 (2,860 – 4,962) | 950 (501 – 1,599) | 0.25 (0.18 – 0.32) |
| 3c | 2008 | 4,366 (3,293 – 5,691) | 3,782 (2,830 – 4,872) | 937 (489 – 1,565) | 0.25 (0.17 – 0.32) |
| 4 | 2008 | 4,330 (3,235 – 5,616) | 3,892 (2,918 – 4,986) | 1,034 (546 – 1,731) | 0.27 (0.19 – 0.35) |
| 5a | 2008 | 4,345 (3,255 – 5,638) | 3,780 (2,839 – 4,891) | 932 (483 – 1,578) | 0.25 (0.17 – 0.32) |
| 5b | 2008 | 4,398 (3,330 – 5,731) | 3,794 (2,875 – 4,919) | 946 (491 – 1,581) | 0.25 (0.17 – 0.32) |
| 6a | 2008 | 4,474 (3,424 – 5,714) | 3,815 (2,899 – 4,876) | 885 (460 – 1,501) | 0.23 (0.16 – 0.31) |
| 6b | 2008 | 4,386 (3,320 – 5,699) | 3,808 (2,880 – 4,908) | 920 (463 – 1,557) | 0.24 (0.16 – 0.32) |
| 7 | 2004 | 4,471 (3,165 – 5,782) | 3,543 (2,448 – 4,576) | 908 (464 – 1,411) | 0.26 (0.19 – 0.31) |
| 8 | 2008 | 4,357 (3,237 – 5,643) | 3,783 (2,818 – 4,875) | 936 (475 – 1,557) | 0.25 (0.17 – 0.32) |
| 9a | 2007 | 4,447 (3,197 – 5,852) | 3,660 (2,520 – 4,869) | 974 (175 – 2,037) | 0.27 (0.07 – 0.42) |
| 9b | 2008 | 4,301 (3,333 – 5,655) | 3,726 (2,877 – 4,877) | 1,059 (611 – 1,797) | 0.28 (0.21 – 0.37) |
| 10a | 2008 | 4,545 (3,087 – 5,462) | 4,011 (2,709 – 4,858) | 3,100 (1,939 – 3,854) | 0.77 (0.72 – 0.79) |
| 10b | 2008 | 4,481 (3,109 – 5,519) | 3,992 (2,710 – 4,853) | 3,217 (2,061 – 3,957) | 0.81 (0.76 – 0.82) |
| 11 | 2008 | 4,338 (3,330 – 5,699) | 3,764 (2,869 – 4,886) | 1,000 (549 – 1,680) | 0.27 (0.19 – 0.34) |
| 12 | 2010 | 4,256 (3,032 – 5,985) | 4,062 (2,916 – 5,523) | 1,220 (627 – 2,235) | 0.30 (0.22 – 0.40) |
| 13 | 2004 | 4,613 (2,985 – 6,699) | 3,750 (2,783 – 4,402) | 1,080 (756 – 1,316) | 0.29 (0.27 – 0.30) |
| 14a | 2008 | 4,418 (3,301 – 5,845) | 3,815 (2,826 – 4,995) | 959 (482 – 1,621) | 0.25 (0.17 – 0.32) |
| 14b | 2003 | 4,370 (3,420 – 5,636) | 3,687 (2,845 – 4,711) | 883 (480 – 1,469) | 0.24 (0.17 – 0.31) |
| 14c | 2008 | 4,337 (3,358 – 5,628) | 3,776 (2,922 – 4,812) | 935 (512 – 1,541) | 0.25 (0.18 – 0.32) |

CrI: Credible interval. NSP: Needle and syringe programmes. OST: Opioid substitution therapy. PWID: People who inject drugs. ^a^ See Table 2 for further details.

*Table 8: Results for the* ***prevalence*** *of chronic hepatitis C in the baseline model, counterfactual scenarios and sensitivity analyses,* ***ex–PWID****.*

| Model number^a^ | Peak prevalence year | Peak prevalence (95% CrI) | Prevalence in 2015 (95% CrI) | Prevalence in 2022 (95% CrI) | Relative change in prevalence, 2022 compared to 2015 |
| --- | --- | --- | --- | --- | --- |
| Baseline | 2007 | 5,490 (4,077 – 7,289) | 4,634 (3,129 – 6,643) | 993 (129 – 3,052) | 0.21 (0.04 – 0.46) |
| 1a | 2007 | 4,843 (3,819 – 6,160) | 3,940 (2,864 – 5,376) | 374 (110 – 1,719) | 0.09 (0.04 – 0.32) |
| 1b | 2007 | 6,286 (4,282 – 8,497) | 5,520 (3,336 – 8,005) | 1,890 (146 – 4,545) | 0.34 (0.04 – 0.57) |
| 2 | 2007 | 5,992 (4,266 – 8,158) | 5,236 (3,352 – 7,515) | 1,583 (149 – 4,097) | 0.30 (0.04 – 0.55) |
| 3a | 2007 | 5,456 (4,057 – 7,314) | 4,626 (3,094 – 6,662) | 967 (128 – 3,112) | 0.21 (0.04 – 0.47) |
| 3b | 2007 | 5,537 (4,069 – 7,354) | 4,701 (3,121 – 6,694) | 1,052 (127 – 3,159) | 0.22 (0.04 – 0.47) |
| 3c | 2007 | 5,490 (4,064 – 7,238) | 4,667 (3,132 – 6,610) | 1,007 (125 – 3,040) | 0.22 (0.04 – 0.46) |
| 4 | 2007 | 5,240 (3,827 – 6,963) | 4,574 (2,981 – 6,512) | 1,113 (137 – 3,203) | 0.24 (0.05 – 0.49) |
| 5a | 2007 | 5,454 (4,015 – 7,218) | 4,631 (3,062 – 6,610) | 987 (123 – 2,993) | 0.21 (0.04 – 0.45) |
| 5b | 2007 | 5,570 (4,133 – 7,320) | 4,714 (3,188 – 6,678) | 1,054 (127 – 3,069) | 0.22 (0.04 – 0.46) |
| 6a | 2007 | 5,650 (4,238 – 7,337) | 4,736 (3,189 – 6,607) | 841 (117 – 2,870) | 0.18 (0.04 – 0.43) |
| 6b | 2007 | 5,552 (4,123 – 7,302) | 4,702 (3,126 – 6,652) | 968 (120 – 3,025) | 0.21 (0.04 – 0.45) |
| 7 | 2007 | 5,800 (4,069 – 7,443) | 5,021 (3,137 – 6,826) | 1,697 (223 – 3,471) | 0.34 (0.07 – 0.51) |
| 8 | 2007 | 5,491 (3,988 – 7,199) | 4,652 (3,051 – 6,575) | 996 (121 – 2,988) | 0.21 (0.04 – 0.45) |
| 9a | 2010 | 6,060 (4,299 – 8,017) | 5,408 (3,547 – 7,456) | 1,560 (198 – 3,478) | 0.29 (0.06 – 0.47) |
| 9b | 2007 | 5,424 (4,158 – 7,251) | 4,572 (3,187 – 6,599) | 824 (148 – 2,849) | 0.18 (0.05 – 0.43) |
| 10a | 2007 | 5,739 (3,878 – 6,921) | 5,105 (2,995 – 6,443) | 4,225 (2,158 – 5,647) | 0.83 (0.72 – 0.88) |
| 10b | 2007 | 5,680 (3,879 – 6,977) | 5,106 (3,027 – 6,510) | 4,547 (2,436 – 5,991) | 0.89 (0.80 – 0.92) |
| 11 | 2007 | 5,482 (4,119 – 7,231) | 4,635 (3,173 – 6,564) | 954 (137 – 2,965) | 0.21 (0.04 – 0.45) |
| 12 | 2011 | 4,490 (2,918 – 7,045) | 4,140 (2,514 – 6,700) | 1,192 (148 – 4,000) | 0.29 (0.06 – 0.60) |
| 13 | 2003 | 5,132 (3,333 – 7,341) | 4,528 (617 – 6,399) | 287 (163 – 523) | 0.06 (0.26 – 0.08) |
| 14a | 2007 | 5,576 (4,083 – 7,427) | 4,739 (3,105 – 6,771) | 1,069 (124 – 3,195) | 0.23 (0.04 – 0.47) |
| 14b | 2007 | 5,590 (4,272 – 7,247) | 4,670 (3,275 – 6,474) | 932 (129 – 2,767) | 0.20 (0.04 – 0.43) |
| 14c | 2007 | 5,479 (4,136 – 7,201) | 4,626 (3,212 – 6,519) | 978 (135 – 2,896) | 0.21 (0.04 – 0.44) |

CrI: Credible interval. NSP: Needle and syringe programmes. OST: Opioid substitution therapy. PWID: People who inject drugs. ^a^ See Table 2 for further details.

*Table 9: Results for the* ***prevalence*** *of chronic hepatitis C in the baseline model, counterfactual scenarios and sensitivity analyses,* ***immigrants****.*

| Model number^a^ | Peak prevalence year | Peak prevalence (95% CrI) | Prevalence in 2015 (95% CrI) | Prevalence in 2022 (95% CrI) | Relative change in prevalence, 2022 compared to 2015 |
| --- | --- | --- | --- | --- | --- |
| Baseline | 2014 | 2,036 (1,854 – 2,186) | 1,995 (1,776 – 2,176) | 1,278 (657 – 1,979) | 0.64 (0.37 – 0.91) |
| 1a | 2014 | 1,974 (1,820 – 2,112) | 1,915 (1,732 – 2,088) | 909 (641 – 1,583) | 0.47 (0.37 – 0.76) |
| 1b | 2014 | 2,103 (1,882 – 2,260) | 2,076 (1,802 – 2,269) | 1,624 (676 – 2,298) | 0.78 (0.37 – 1.01) |
| 2 | 2014 | 2,081 (1,880 – 2,237) | 2,048 (1,803 – 2,237) | 1,525 (673 – 2,214) | 0.74 (0.37 – 0.99) |
| 3a | 2014 | 2,034 (1,857 – 2,194) | 1,989 (1,778 – 2,186) | 1,271 (658 – 1,992) | 0.64 (0.37 – 0.91) |
| 3b | 2014 | 2,039 (1,864 – 2,192) | 1,998 (1,781 – 2,185) | 1,304 (659 – 1,993) | 0.65 (0.37 – 0.91) |
| 3c | 2014 | 2,035 (1,852 – 2,189) | 1,991 (1,772 – 2,177) | 1,281 (652 – 1,971) | 0.64 (0.37 – 0.91) |
| 4 | 2014 | 2,023 (1,826 – 2,180) | 1,979 (1,750 – 2,174) | 1,331 (661 – 2,003) | 0.67 (0.38 – 0.92) |
| 5a | 2014 | 2,034 (1,851 – 2,188) | 1,988 (1,768 – 2,185) | 1,272 (654 – 1,974) | 0.64 (0.37 – 0.90) |
| 5b | 2014 | 2,044 (1,867 – 2,196) | 2,003 (1,781 – 2,177) | 1,305 (654 – 1,987) | 0.65 (0.37 – 0.91) |
| 6a | 2014 | 1,753 (1,596 – 1,895) | 1,706 (1,523 – 1,874) | 1,030 (565 – 1,664) | 0.60 (0.37 – 0.89) |
| 6b | 2014 | 1,994 (1,820 – 2,148) | 1,949 (1,742 – 2,134) | 835 (330 – 1,503) | 0.43 (0.19 – 0.70) |
| 7 | 2015 | 3,552 (3,158 – 3,835) | 3,552 (3,158 – 3,835) | 2,420 (921 – 3,434) | 0.68 (0.29 – 0.90) |
| 8 | 2014 | 2,037 (1,841 – 2,192) | 1,994 (1,755 – 2,174) | 1,285 (655 – 1,952) | 0.64 (0.37 – 0.90) |
| 9a | 2014 | 2,244 (2,087 – 2,357) | 2,231 (2,045 – 2,368) | 1,591 (861 – 2,037) | 0.71 (0.42 – 0.86) |
| 9b | 2014 | 2,030 (1,863 – 2,193) | 1,984 (1,783 – 2,181) | 1,152 (657 – 1,833) | 0.58 (0.37 – 0.84) |
| 10a | 2022 | 2,466 (2,062 – 2,679) | 2,089 (1,836 – 2,232) | 2,466 (2,062 – 2,679) | 1.18 (1.12 – 1.20) |
| 10b | 2022 | 2,604 (2,192 – 2,786) | 2,114 (1,857 – 2,247) | 2,604 (2,192 – 2,786) | 1.23 (1.18 – 1.24) |
| 11 | 2014 | 2,036 (1,864 – 2,188) | 1,991 (1,787 – 2,180) | 1,239 (661 – 1,925) | 0.62 (0.37 – 0.88) |
| 12 | 2014 | 1,952 (1,715 – 2,178) | 1,908 (1,622 – 2,176) | 1,338 (660 – 2,178) | 0.70 (0.41 – 1.00) |
| 13 | 2016 | 2,346 (312 – 2,814) | 2,232 (319 – 2,686) | 681 (634 – 884) | 0.30 (1.99 – 0.33) |
| 14a | 2014 | 2,045 (1,858 – 2,202) | 2,004 (1,774 – 2,200) | 1,319 (658 – 2,032) | 0.66 (0.37 – 0.92) |
| 14b | 2014 | 2,038 (1,874 – 2,182) | 1,994 (1,800 – 2,175) | 1,256 (659 – 1,902) | 0.63 (0.37 – 0.87) |
| 14c | 2014 | 2,035 (1,864 – 2,180) | 1,994 (1,787 – 2,169) | 1,271 (661 – 1,929) | 0.64 (0.37 – 0.89) |

CrI: Credible interval. NSP: Needle and syringe programmes. OST: Opioid substitution therapy. PWID: People who inject drugs. ^a^ See Table 2 for further details.

*Table 10: Results for the* ***incidence*** *of hepatitis C among* ***active PWID*** *in the baseline model, counterfactual scenarios and sensitivity analyses.*

| Model number^a^ | Peak incidence year | Peak incidence (95% CrI) | Incidence in 2015 (95% CrI) | Incidence per 100 in 2015 (95% CrI) | Incidence in 2022 (95% CrI) | Incidence per 100 in 2022 (95% CrI) | Relative change, 2022 compared to 2015 | Incidence in 2030 (95% CrI) | Incidence per 100 in 2030 (95% CrI) | Relative change, 2030 compared to 2015 |
| --- | --- | --- | --- | --- | --- | --- | --- | --- | --- | --- |
| baseline | 2000 | 726 (506 – 1,067) | 144 (109 – 176) | 1.72 (1.33 – 2.10) | 30 (13 – 52) | 0.37 (0.17 – 0.65) | 0.21 (0.12 – 0.30) | 8 (0 – 52) | 0.11 (0.00 – 0.38) | 0.06 (0.00 – 0.17) |
| 1a | 2000 | 656 (486 – 911) | 120 (94 – 143) | 1.69 (1.33 – 2.01) | 22 (10 – 39) | 0.33 (0.16 – 0.56) | 0.18 (0.11 – 0.27) | 3 (0 – 39) | 0.05 (0.00 – 0.26) | 0.03 (0.00 – 0.12) |
| 1b | 1999 | 819 (537 – 1,201) | 168 (122 – 205) | 1.76 (1.28 – 2.12) | 39 (16 – 67) | 0.43 (0.18 – 0.72) | 0.23 (0.13 – 0.33) | 15 (1 – 67) | 0.17 (0.01 – 0.49) | 0.09 (0.01 – 0.22) |
| 2 | 2000 | 816 (534 – 1,216) | 149 (114 – 180) | 1.78 (1.39 – 2.16) | 34 (15 – 60) | 0.44 (0.19 – 0.76) | 0.23 (0.13 – 0.33) | 13 (1 – 60) | 0.17 (0.01 – 0.52) | 0.09 (0.01 – 0.22) |
| 3a | 2000 | 725 (503 – 1,072) | 143 (109 – 175) | 1.72 (1.32 – 2.08) | 29 (14 – 52) | 0.37 (0.18 – 0.64) | 0.20 (0.13 – 0.30) | 8 (0 – 52) | 0.11 (0.00 – 0.38) | 0.06 (0.00 – 0.17) |
| 3b | 2000 | 734 (515 – 1,064) | 142 (109 – 174) | 1.71 (1.32 – 2.09) | 30 (13 – 52) | 0.38 (0.17 – 0.65) | 0.21 (0.12 – 0.30) | 8 (0 – 52) | 0.11 (0.00 – 0.40) | 0.06 (0.00 – 0.18) |
| 3c | 2000 | 725 (503 – 1,058) | 142 (109 – 175) | 1.71 (1.31 – 2.07) | 29 (14 – 54) | 0.36 (0.18 – 0.66) | 0.20 (0.13 – 0.31) | 8 (0 – 54) | 0.10 (0.00 – 0.37) | 0.06 (0.00 – 0.17) |
| 4 | 2000 | 728 (496 – 1,030) | 182 (145 – 217) | 2.18 (1.76 – 2.59) | 47 (22 – 77) | 0.59 (0.28 – 0.95) | 0.26 (0.15 – 0.35) | 15 (1 – 77) | 0.20 (0.02 – 0.62) | 0.08 (0.01 – 0.23) |
| 5a | 2000 | 723 (490 – 1,041) | 146 (112 – 178) | 1.75 (1.34 – 2.11) | 30 (13 – 52) | 0.38 (0.17 – 0.65) | 0.21 (0.12 – 0.29) | 8 (0 – 52) | 0.11 (0.00 – 0.39) | 0.05 (0.00 – 0.17) |
| 5b | 2000 | 741 (508 – 1,064) | 139 (106 – 169) | 1.67 (1.28 – 2.02) | 28 (12 – 51) | 0.36 (0.16 – 0.63) | 0.20 (0.11 – 0.30) | 7 (0 – 51) | 0.10 (0.00 – 0.37) | 0.05 (0.00 – 0.17) |
| 6a | 2000 | 765 (539 – 1,082) | 148 (116 – 179) | 1.78 (1.39 – 2.13) | 30 (13 – 54) | 0.37 (0.17 – 0.66) | 0.20 (0.11 – 0.30) | 7 (0 – 54) | 0.09 (0.00 – 0.36) | 0.05 (0.00 – 0.16) |
| 6b | 2000 | 739 (502 – 1,071) | 145 (113 – 176) | 1.74 (1.36 – 2.10) | 30 (14 – 53) | 0.38 (0.18 – 0.66) | 0.21 (0.12 – 0.30) | 6 (0 – 53) | 0.09 (0.00 – 0.34) | 0.04 (0.00 – 0.15) |
| 7 | 2000 | 824 (548 – 1,143) | 170 (125 – 210) | 2.04 (1.51 – 2.49) | 37 (17 – 63) | 0.46 (0.22 – 0.78) | 0.22 (0.14 – 0.30) | 10 (1 – 63) | 0.14 (0.01 – 0.38) | 0.06 (0.01 – 0.14) |
| 8 | 2000 | 728 (491 – 1,045) | 143 (110 – 175) | 1.72 (1.32 – 2.08) | 29 (13 – 53) | 0.37 (0.16 – 0.66) | 0.20 (0.12 – 0.30) | 8 (0 – 53) | 0.11 (0.00 – 0.36) | 0.06 (0.00 – 0.16) |
| 9a | 2000 | 780 (518 – 1,113) | 138 (101 – 171) | 1.66 (1.21 – 2.03) | 28 (6 – 64) | 0.36 (0.08 – 0.78) | 0.20 (0.06 – 0.37) | 12 (0 – 64) | 0.17 (0.00 – 0.61) | 0.09 (0.00 – 0.29) |
| 9b | 2000 | 718 (511 – 1,040) | 142 (110 – 174) | 1.71 (1.32 – 2.07) | 33 (16 – 59) | 0.42 (0.21 – 0.72) | 0.23 (0.15 – 0.34) | 9 (1 – 59) | 0.12 (0.01 – 0.43) | 0.06 (0.01 – 0.20) |
| 10a | 2000 | 769 (441 – 1,008) | 148 (111 – 182) | 1.78 (1.31 – 2.16) | 76 (48 – 98) | 0.97 (0.61 – 1.25) | 0.51 (0.43 – 0.54) | 58 (33 – 98) | 0.82 (0.47 – 1.17) | 0.39 (0.29 – 0.47) |
| 10b | 2000 | 754 (457 – 997) | 145 (107 – 179) | 1.72 (1.27 – 2.12) | 77 (53 – 100) | 0.98 (0.67 – 1.27) | 0.53 (0.50 – 0.56) | 62 (38 – 100) | 0.88 (0.53 – 1.19) | 0.43 (0.35 – 0.48) |
| 11 | 2000 | 727 (504 – 1,049) | 143 (111 – 172) | 1.71 (1.33 – 2.06) | 33 (16 – 57) | 0.41 (0.20 – 0.70) | 0.23 (0.14 – 0.33) | 8 (0 – 57) | 0.10 (0.00 – 0.37) | 0.06 (0.00 – 0.17) |
| 12 | 2000 | 743 (485 – 1,151) | 271 (225 – 311) | 3.26 (2.74 – 3.72) | 89 (50 – 141) | 1.11 (0.64 – 1.70) | 0.33 (0.22 – 0.45) | 37 (6 – 141) | 0.50 (0.08 – 1.40) | 0.14 (0.03 – 0.37) |
| 13 | 2000 | 825 (485 – 1,343) | 182 (140 – 220) | 2.17 (1.69 – 2.61) | 52 (35 – 71) | 0.67 (0.44 – 0.91) | 0.29 (0.25 – 0.32) | 9 (3 – 71) | 0.13 (0.04 – 0.28) | 0.05 (0.02 – 0.09) |
| 14a | 2000 | 746 (504 – 1,091) | 146 (110 – 176) | 1.75 (1.33 – 2.10) | 31 (14 – 56) | 0.38 (0.18 – 0.69) | 0.21 (0.13 – 0.32) | 9 (0 – 56) | 0.12 (0.00 – 0.42) | 0.06 (0.00 – 0.19) |
| 14b | 1999 | 800 (566 – 1,118) | 166 (126 – 203) | 1.98 (1.51 – 2.39) | 33 (15 – 60) | 0.41 (0.20 – 0.74) | 0.20 (0.12 – 0.30) | 8 (1 – 60) | 0.12 (0.01 – 0.43) | 0.05 (0.01 – 0.16) |
| 14c | 2000 | 725 (512 – 1,042) | 144 (111 – 176) | 1.73 (1.34 – 2.10) | 30 (13 – 51) | 0.37 (0.17 – 0.64) | 0.21 (0.12 – 0.29) | 8 (0 – 51) | 0.11 (0.00 – 0.36) | 0.06 (0.00 – 0.16) |

CrI: Credible interval. NSP: Needle and syringe programmes. OST: Opioid substitution therapy. PWID: People who inject drugs. ^a^ See Table 2 for further details.

# References

1. FitzJohn R BM, Knock E, Whittles L, Lees J, Sonabend R. mcstate: Monte Carlo Methods for State Space Models. R package version 0.9.17. 2023. Available at: <https://github.com/mrc-ide/mcstate>. Accessed 15 November 2023.

2. FitzJohn RG, Knock ES, Whittles LK, Perez-Guzman PN, Bhatia S, Guntoro F, et al. Reproducible parallel inference and simulation of stochastic state space models using odin, dust, and mcstate. Wellcome Open Res. 2020;5:288.

3. Meijerink H, White RA, Løvlie A, de Blasio BF, Dalgard O, Amundsen EJ, et al. Modelling the burden of hepatitis C infection among people who inject drugs in Norway, 1973-2030. BMC Infect Dis. 2017;17(1):541.

4. Statistics Norway. 05381: Deaths, by sex and age (per 100 000 mean population) 1976 – 2022. 2023. Available at: <https://www.ssb.no/en/statbank/table/05381/>. Accessed 15 November 2023. License for data use available at: [Licence (ssb.no)](https://www.ssb.no/en/diverse/lisens).

5. The Norwegian Institute of Public Health. Overdosedødsfall i 2022 [In Norwegian]. 2023. Available at: <https://www.fhi.no/nyheter/2023/stabil-trend-flere-overdosedodsfall-enn-i-2021-men-farre-enn-i-2020/#:~:text=Det%20totale%20antallet%20narkotikautl%C3%B8ste%20d%C3%B8dsfall,p%C3%A5%20280%20d%C3%B8dsfall%20per%20%C3%A5r>. Accessed 15 November 2023.

6. Amundsen EJ, Bretteville-Jensen AL, Kraus L. Estimating incidence of problem drug use using the Horwitz-Thompson estimator - A new approach applied to people who inject drugs in Oslo 1985-2008. Int J Drug Policy. 2016;27:36-42.

7. The Norwegian Institute of Public Health. Problembruk av narkotika [In Norwegian]. 2022. Available at: <https://www.fhi.no/nettpub/narkotikainorge/bruk-av-narkotika/problembruk-av-narkotika/?term=&h=1>. Accessed 15 November 2023.

8. Centre for Alcohol and Drug Research. Utdeling av utstyr for skadereduksjon ved rusmiddelbruk Kommuneundersøkelse 2020 [In Norwegian]. 2020. Available at: <https://www.helse-stavanger.no/4ac81c/siteassets/seksjon/korfor/documents/brukerplan/brukerutstyrsundersokelse-2020.pdf>. Accessed 15 November 2023.

9. Norwegian Cause of Death Registry. Dødsårsaksregisteret - statistikkbank: Tabell D7 [In Norwegian]. 2023. Available at: <http://statistikkbank.fhi.no/dar/>. Accessed 15 November 2023.

10. Statistics Norway. Immigrants and Norwegian-born to immigrant parents, tabell 05184: Immigrants, by sex and country background 1970 – 2023. 2023. Available at: <https://www.ssb.no/en/statbank/table/05184/>. Accessed 15 November 2023. License for data use available at: [Licence (ssb.no)](https://www.ssb.no/en/diverse/lisens).

11. Polaris Observatory HCV Collaborators. Global change in hepatitis C virus prevalence and cascade of care between 2015 and 2020: a modelling study. Lancet Gastroenterol Hepatol. 2022;7(5):396-415.

12. Nordoy I, Krarup HB, Bell H, Christensen PB, Elgjo K, von der Lippe B, et al. Interferon-alpha 2b therapy in low-activity hepatitis C: a pilot study. Scand J Gastroenterol. 1997;32(12):1256-60.

13. Bell H, Hellum K, Harthug S, Myrvang B, Ritland S, Maeland A, et al. Treatment with interferon-alpha2a alone or interferon-alpha2a plus ribavirin in patients with chronic hepatitis C previously treated with interferon-alpha2a. CONSTRUCT Group. Scand J Gastroenterol. 1999;34(2):194-8.

14. Bjoro K, Bell H, Hellum KB, Skaug K, Raknerud N, Sandvei P, et al. Effect of combined interferon-alpha induction therapy and ribavirin on chronic hepatitis C virus infection: a randomized multicentre study. Scand J Gastroenterol. 2002;37(2):226-32.

15. The Norwegian Directorate of eHealth. Norwegian Prescribed Drug Registry. 2023. Available at: <https://helsedata.no/en/forvaltere/norwegian-institute-of-public-health/norwegian-prescribed-drug-registry/>. Accessed 15 November 2023.

16. WHO Collaborating Centre for Drug Statistics Methodology. ATC/DDD Index 2023. 2023. Available at: <https://www.whocc.no/atc_ddd_index/>. Accessed 15 November 2023.

17. Dalgard O, Konopski Z, Bosse FJ, Nordstrand B, Sandvei P, Karlsen L, et al. Hepatitt C – utredning og behandling [In Norwegian]. Tidsskr Nor Legeforen. 2011. doi:10.4045/tidsskr.10.02401

18. Norwegian Medical Association. Faglig veileder for utredning og behandling av hepatitt C [In Norwegian]. 2014. Available at: <https://gastroenterologen.no/2014/10/faglig-veileder-forutredning-og-behandling-av-hepatitt-c/>. Accessed 15 November 2023.

19. Norwegian Medical Association. Faglig veileder for utredning og behandling av hepatitt C [In Norwegian]. 2015. Available at: <https://www.legeforeningen.no/contentassets/679b162da45e41e9b742010021e6ff89/2015-09-22-hcv-veileder-revisjon-sept-2015rev.pdf>. Accessed 15 November 2023.

20. Norwegian Medical Association. Faglig veileder for utredning og behandling av hepatitt C hos voksne [In Norwegian]. 2017. Available at: <https://www.legeforeningen.no/contentassets/4e9314a6e12047b791186762ebc174b8/hepatitt-c-veileder-200317.pdf>. Accessed 15 November 2023.

21. Norwegian Medical Association. Faglig veileder for utredning og behandling av hepatitt C hos voksne [In Norwegian]. 2019. Available at: <https://www.legeforeningen.no/contentassets/be28359cd11745a8bbc090e93d9b9c90/veileder-revisjon-hcv.pdf>. Accessed 15 November 2023.

22. Norwegian Medical Association. Faglig veileder for utredning og behandling av hepatitt C [In Norwegian]. 2022. Available at: <https://www.legeforeningen.no/contentassets/7e304300c0b5464eaf682fa7b70dd659/veileder-hepatitt-c-2022.pdf>. Accessed 15 November 2023.

23. Kristiansen MG, Gutteberg T, Berg LK, Sjursen H, Mortensen L, Florholmen J. Hepatitt C i Nord-Norge – et åtteårsmateriale [In Norwegian]. Tidsskr Nor Legeforen. 2002;122(20):1974-6.

24. Dalgard O, Jeansson S, Skaug K, Raknerud N, Bell H. Hepatitis C in the general adult population of Oslo: prevalence and clinical spectrum. Scand J Gastroenterol. 2003;38(8):864-70.

25. Kileng H, Gutteberg T, Goll R, Paulssen EJ. Screening for hepatitis C in a general adult population in a low-prevalence area: the Tromso study. BMC Infect Dis. 2019;19(1):189.

26. Malme KB, Ulstein K, Finbråten AK, Wüsthoff LEC, Kielland KB, Hauge J, et al. Hepatitis C treatment uptake among people who inject drugs in Oslo, Norway: A registry-based study. Int J Drug Policy. 2023;116:104044.

27. Kristiansen MG, Lochen ML, Gutteberg TJ, Mortensen L, Eriksen BO, Florholmen J. Total and cause-specific mortality rates in a prospective study of community-acquired hepatitis C virus infection in northern Norway. J Viral Hepat. 2011;18(4):237-44.

28. Isaksen K, Aabakken L, Grimstad T, Karlsen L, Sandvei PK, Dalgard O. Hepatitis C treatment at three Norwegian hospitals 2000-2011. Tidsskr Nor Legeforen. 2015;135(22):2052-8.

29. The Norwegian Institute of Public Health. Statusrapport om eliminasjon av hepatitt B og C som folkehelseproblem i Norge: Oppfølging av den nasjonale strategien mot virale hepatitter [In Norwegian]. 2023. Available at: <https://www.fhi.no/publ/2023/statusrapport-om-eliminasjon-av--hepatitt-b-og-c-som--folkehelseproblem-i-n/>. Accessed 15 November 2023.

30. Manns MP, Maasoumy B. Breakthroughs in hepatitis C research: from discovery to cure. Nat Rev Gastroenterol Hepatol. 2022;19(8):533-50.

31. Hezode C, Forestier N, Dusheiko G, Ferenci P, Pol S, Goeser T, et al. Telaprevir and peginterferon with or without ribavirin for chronic HCV infection. N Engl J Med. 2009;360(18):1839-50.

32. Poordad F, McCone J, Jr., Bacon BR, Bruno S, Manns MP, Sulkowski MS, et al. Boceprevir for untreated chronic HCV genotype 1 infection. N Engl J Med. 2011;364(13):1195-206.

33. Zoratti MJ, Siddiqua A, Morassut RE, Zeraatkar D, Chou R, van Holten J, et al. Pangenotypic direct acting antivirals for the treatment of chronic hepatitis C virus infection: A systematic literature review and meta-analysis. EClinicalMedicine. 2020;18:100237.

34. Norton BL, Akiyama MJ, Arnsten JH, Agyemang L, Heo M, Litwin AH. High HCV cure rates among people who inject drugs and have suboptimal adherence: A patient-centered approach to HCV models of care. Int J Drug Policy. 2021;93:103135.

35. Cunningham EB, Hajarizadeh B, Amin J, Litwin AH, Gane E, Cooper C, et al. Adherence to Once-daily and Twice-daily Direct-acting Antiviral Therapy for Hepatitis C Infection Among People With Recent Injection Drug Use or Current Opioid Agonist Therapy. Clin Infect Dis. 2020;71(7):e115-e24.

36. Ministry of Health and Care Services. Nasjonal strategi mot hepatitter [In Norwegian]. 2018. Available at: <https://www.regjeringen.no/no/dokumenter/nasjonal-strategi-mot-hepatitter/id2618584/>. Accessed 15 November 2023.

37. Midgard H, Ulstein K, Backe Ø, Foshaug T, Sørli H, Vennesland K, et al. Hepatitis C treatment and reinfection surveillance among people who inject drugs in a low-threshold program in Oslo, Norway. Int J Drug Policy. 2021;96:103165.

38. Hajarizadeh B, Cunningham EB, Reid H, Law M, Dore GJ, Grebely J. Direct-acting antiviral treatment for hepatitis C among people who use or inject drugs: a systematic review and meta-analysis. Lancet Gastroenterol Hepatol. 2018;3(11):754-67.

39. Graf C, Mucke MM, Dultz G, Peiffer KH, Kubesch A, Ingiliz P, et al. Efficacy of Direct-acting Antivirals for Chronic Hepatitis C Virus Infection in People Who Inject Drugs or Receive Opioid Substitution Therapy: A Systematic Review and Meta-analysis. Clin Infect Dis. 2020;70(11):2355-65.

40. Micallef JM, Kaldor JM, Dore GJ. Spontaneous viral clearance following acute hepatitis C infection: a systematic review of longitudinal studies. J Viral Hepat. 2006;13(1):34-41.

41. Platt L, Minozzi S, Reed J, Vickerman P, Hagan H, French C, et al. Needle and syringe programmes and opioid substitution therapy for preventing HCV transmission among people who inject drugs: findings from a Cochrane Review and meta-analysis. Addiction. 2018;113(3):545-63.

42. Norwegian Centre for Addiction Research. Statusrapport 2022 [In Norwegian]. 2023. Available at: <https://www.med.uio.no/klinmed/forskning/sentre/seraf/publikasjoner/rapporter/2023/seraf-rapport-nr-1-2023-statusrapport-2022.html>. Accessed 15 November 2023.

43. Hannula R, Soderholm J, Svendsen T, Skaland M, Nordbo SA, Steinum H, et al. Hepatitis C outreach project and cross-sectional epidemiology in high-risk populations in Trondheim, Norway. Ther Adv Infect Dis. 2021;8:20499361211053929.
